# Key determinants to supply chain resilience to face pandemic disruption: An interpretive triple helix framework

**DOI:** 10.1371/journal.pone.0299778

**Published:** 2024-05-01

**Authors:** Md. Galib An-Noor Amio, Humaira Nafisa Ahmed, Syed Mithun Ali, Sayem Ahmed, Abhijit Majumdar

**Affiliations:** 1 Department of Industrial and Production Engineering, Bangladesh University of Engineering and Technology, Dhaka, Bangladesh; 2 Department of Mechanical and Production Engineering, Ahsanullah University of Science and Technology, Dhaka, Bangladesh; 3 Department of Textile and Fibre Engineering, Indian Institute of Technology, Delhi, India; Gonbad Kavous University, ISLAMIC REPUBLIC OF IRAN

## Abstract

Today, supply chain (SC) networks are facing more disruptions compared to the past. While disruptions are rare, they can have catastrophic long-term economic or societal repercussions, and the recovery processes can be lengthy. These can tremendously affect the SC and make it vulnerable, as observed during the COVID-19 pandemic. The identification of these concerns has prompted the demand for improved disruption management by developing resilient, agile, and adaptive SC. The aim of this study is to introduce an assessment framework for prioritizing and evaluating the determinants to supply chain resilience (SCR). To analyze the empirical data, fuzzy criteria importance through intercriteria correlation (fuzzy CRITIC) and fuzzy technique for order of preference by similarity to ideal solution (fuzzy TOPSIS) have been incorporated. Fuzzy CRITIC method was used to identify the critical determinants and fuzzy TOPSIS method was applied for determining relative ranking of some real-world companies. Finally, by developing propositions an interpretive triple helix framework was proposed to achieve SCR. This research stands out for its originality in both methodology and implications. By introducing the novel combination of Fuzzy CRITIC and Fuzzy TOPSIS in the assessment of determinants to SCR and applying these determinants with the help of interpretive triple helix framework to establish a resilient SC, this study offers a unique and valuable contribution to the field of SCR. The key findings suggest that ‘Responsiveness’ followed by ‘Managerial coordination and information integration’ are the most significant determinant to achieve SCR. The outcome of this work can assist the managers to achieve SCR with improved agility and adaptivity.

## 1. Introduction

Industries nowadays are getting increasingly concerned about becoming more efficient, subsequently lacking the supply chain resilience (SCR) culture [1]. Resilience in supply chain (SC) refers to the ability of a system to remain robust and modify its behavior in dynamic contexts in the event of significant disruptions while maintaining acceptable performance [2–5]. However, increased globalization and uncertainty in dynamic contexts make the overall SC scenario riskier and more vulnerable where industries are also not prepared to deal with sudden disruptions. Ideally, SC should recover as rapidly as feasible from disruption in order to maintain operational continuity and its competitive edge. The concept of resilience can be characterized as the capacity to strategically plan and take proactive measures for anticipating unforeseen disruptive events and the ability to adapt and respond while preserving control over the critical functions [6, 7]. Recently, studies have demonstrated the importance of SCR in light of recent disruptions, such as the ongoing global epidemic caused by COVID-19 [8].

The effects of SC disruptions can be more clearly seen in manufacturing firms, healthcare institutions, and hospitality industries such as- hotel, restaurant, and travel industries [9]. SCs are now prone to disruptions due to the risks induced by internal and external factors. External factors include globalization, supply network complexity, uncertainty in demand and supply; and the internal factors include the need for agility, the growing practice of lean manufacturing practices, and just-in-time production processes [10, 11]. Resilience is claimed to play a major role in restoring operations and limiting risk, although the approaches and methods for achieving resilience have not been thoroughly investigated. In the past decade, the SC network has gotten more advanced due to the attempts made to drastically cut costs and make it more efficient and leaner. However, the complexity is increasing with the change of competitive environment, usage of advanced innovation, quick globalization, and so on. These are making the SCs less resilient and prone to unforeseen disruptions as evident from the recent global pandemic [8]. The aforementioned discussion demonstrate that disruptions may significantly affect a company’s SC performance, especially in highly uncertain markets; hence, determinants of SCR are vital for successfully surviving any sudden disaster.

Managing the SC networks to make it more resilient in order to have the ability to withstand and minimize loss or perhaps avoid it altogether has thus obtained a greater research interest. The performance and profitability of business organizations are also largely dependent on the success of their SC especially during unplanned disruptions such as COVID-19 [12–14]. Therefore, for a deeper understanding and future development of network-based supply concepts from a cross-disciplinary viewpoint, methodical elaborations on the evaluation and understanding of determinants supporting SCR are essential. The purpose of this research is to determine the major determinants related to SCR where the policymakers can concentrate and intervene, also, to develop an assessment framework for evaluating the determinants and prioritizing them in order of importance and measuring the SCR performance of companies. Additionally, this work serves to answer the following research questions (RQs).

***RQ1*.** What are the critical determinants that should be prioritized in order to achieve SCR?

***Motivation for RQ1*:** There was a lack of comprehensive understanding regarding the specific factors or determinants that significantly contribute to SCR. While the importance of resilience was acknowledged, the specific elements that bolster it were not clearly defined. Answering this question will list the critical determinants in achieving SCR. By understanding the determinants, companies can strengthen key areas and develop robust systems that can withstand and recover from disruptions.

***RQ2*.** How can these determinants facilitate policymakers in achieving SCR?

***Motivation for RQ2*:** Policymakers often recognize certain elements crucial to resilience, but the formal identification and categorization of these factors as determinants might be lacking. While policymakers possess practical insights into factors contributing to resilience, a formal and structured identification of these elements as ’determinants’ might be an area that requires more deliberate attention and systematic analysis. Answering this question will give information on how to analyze and prioritize those determinants and compare this result with the results obtained from previous literature. Understanding and acting upon these determinants allow companies to distinguish themselves by ensuring stability, customer satisfaction, and market positioning even in times of crisis.

***RQ3*.** How cross-disciplinary approaches will enable organizations to establish SCR?

***Motivation for RQ3*:** Industry-centric approaches might be limited to immediate operational concerns, while academia’s focus on theoretical frameworks that might sometimes lack practical applicability or fail to address the immediate needs of industries. Government actions can sometimes face bureaucratic challenges, delaying policy formulation or implementation, especially during urgent situations. Moreover, government policies might be formulated without extensive consultation or collaboration with industry stakeholders, limiting their relevance and effectiveness. Thus, approaching resilience from one sector’s viewpoint might overlook critical aspects or interconnected issues affecting the broader SC system. Addressing this question will tackle the challenges encountered by various stakeholders and offer propositions for effectively managing them in order to successfully implement SCR.

An integrated approach, composed of fuzzy criteria importance through intercriteria correlation (fuzzy CRITIC) and fuzzy technique for order of preference by similarity to ideal solution (fuzzy TOPSIS) method has been employed for the evaluation of the determinants to find their relative weights in order to prioritize them as of importance and rank the companies based on the determinants. Fuzzy CRITIC and Fuzzy TOPSIS offer several notable advantages in the realm of multi-criteria decision-making (MCDM) when compared to more recent methods like MEREC (Method based on the Removal Effects of Criteria), SECA (Simultaneous Evaluation of Criteria and Alternatives), VIKOR (VIseKriterijumska Optimizacija I Kompromisno Resenje) and EDAS (Evaluation based on Distance from Average Solution). Fuzzy CRITIC and Fuzzy TOPSIS excel in handling uncertainty and vagueness, making it a valuable choice for real-world decision problems with imprecise information [15]. Unlike MEREC, Fuzzy CRITIC considers intercriteria correlations which can more closely mirror the actual SC environment, providing a more nuanced perspective on criteria importance [16]. In contrast, traditional SECA and EDAS may not effectively handle qualitative or imprecise data, potentially restricting its applicability in situations where such information is prevalent. Additionally, SECA and VIKOR may face computational complexity challenges when managing an extensive array of criteria and alternatives, potentially making it less practical in certain scenarios [17]. On the other hand, Fuzzy TOPSIS effectively balances both optimistic and pessimistic approach in decision-making by finding a compromise solution, in contrast to VIKOR and EDAS for being overly optimistic and more pessimistic, respectively. Also, it ranks alternatives based on their relative distances to ideal and anti-ideal solutions, providing a geometric perspective on performance evaluation [18]. The transparent and intuitive approach of Fuzzy CRITIC and Fuzzy TOPSIS enable decision-makers to articulate their judgments using linguistic terms, promoting better understanding [19]. The choice among these methods ultimately depends on the specific characteristics and data available in the decision problem. Adaptability, robustness, and holistic evaluation of Fuzzy CRITIC and Fuzzy TOPSIS make them flexible and comprehensive MCDM tool for evaluating the ranking of companies [18].

Identifying determinants for assessing SCR involves analyzing various aspects that contribute to an organization’s capability to withstand and recover quickly from disruptions. Ranking companies based on these determinants in a situation like the COVID-19 pandemic would involve assessing how each company responded in terms of these factors during the crisis. Cross-disciplinary approaches are essential for addressing the complexities inherent in SCR. By integrating insights and methods from diverse fields, organizations can better prepare, respond, and adapt to the multifaceted challenges of modern SC. Thus, propositions to achieve the determinants have been developed using the interpretive triple helix framework. The interpretive triple helix framework can be defined as “the evolutionary perspective facilitates the generation of a knowledge based infrastructure overlying the different institutional spheres, where each takes on the role of the other within the framework of an emerging tripartite interface between hybrid organizations” [20]. In this research, the interpretative triple helix paradigm combines the viewpoints of government, industry, and academia to develop strategies for facilitating SCR through the use of determinants. Collaboration among industry, academia, and government facilitates the exchange of knowledge and expertise. Academia contributes cutting-edge research, industry offers practical experience, and government provides policy insights. This interdisciplinary collaboration fosters innovation and the development of novel approaches to enhance SCR. Based on the above discussion, the specific objectives of this study can be stated as follows.

- To identify and prioritize the determinants relevant to SCR and assist policymakers in decision making.
- To analyze the determinants of SCR using fuzzy CRITIC method and evaluate the resilience environment of companies with fuzzy TOPSIS method.
- To develop propositions for the organizations using interpretive triple helix framework.

Six sections comprise the study, where the first section discusses impacts of disruption in SC and the necessity of SCR, section 2 summarizes the review of literature on determinants related to SCR and existing solution approaches. Section 3 describes a structural framework on fuzzy CRITIC and fuzzy TOPSIS. Section 4 analyzes and discusses the results and key findings. Section 5 highlights the implications of the study, while section 6 addresses the study’s scope and limitations.

## 2. Literature review

### 2.1 Supply chain resilience

Christopher & Peck [21] defined resilience as the capacity of a company to survive a disturbance while returning to its original state or even having a transition towards a more favorable state. A more extensive definition of SCR is given by Kamalahmadi & Parast [22], according to them SCR is the capacity of a SC to minimize the likelihood of encountering unexpected disruptions, resist the spread of disruptions by retaining control over processes and functions, recover by implementing immediate reactive plans and finally return the SC to a stable state of operations. Enhancing resilience can be a strategic priority that anticipates systemic reactions to disruptions and re-invents operating models as conditions change, or it can be a tactical initiative that is limited to resources and competencies to recover from a disturbance by re-establishing basic business operations. Evidently resilient organizations are better equipped to manage disruptions and gain sustainable competitive advantage from disruptive events.

Sources of SC risks along with their probabilities and impacts were listed by [23, 24] in the form of a Cartesian coordinate map using a 2×2 matrix. They found political instability, labor unrest, economic downturn, port closures, loss of key suppliers, disruption of major transportation routes to be the highly probable causes of sudden disruptions in SC. These findings were backed up and echoed by several other researchers [25–27]. Various approaches have been suggested in the literature to increase SCR while minimizing the risks. For instance, Adobor & McMullen [28] provided insights on enhancing SCR using approaches such as ecology, engineering along with evolution while recognizing the need of being effective, adaptive as well as having the ability to transform in disruption. Gunasekaran et al. [29] summarized the strategies of achieving SCR in global sourcing to overcome the complexities and emphasized the fact that resilience should also take into consideration complexity factors in order to achieve a holistic outcome rather than only focusing on proactive approaches [29]. According to Pettit et al. [30], in order to achieve effective SCR, an organization needs to establish the ‘four Rs’, namely robustness, resourcefulness, recovery, and review. Although there are a number of organizational activities that can improve a firm’s sensitivity to SC disruptions, the literature identifies redundancy, rerouting, restoration, coordination, visibility, adaptability, and agility as the most critical organizational skills for improving a firm’s responsiveness to SC disruptions [31, 32]. Calvo et al. [33] in a theoretical literature review of SCR indicated the taxonomy of resilient SC demonstrated by Christopher & Peck [21] and argued that there are two fundamental perspectives to resiliency in the SC. They showed that according to the perspective of Sheffi [24] organizations can establish resilience by increasing redundancy, improving the speed of recovery, and shifting of the organizational culture. Another fundamental perspective established by Christopher & Towill [34] talks about establishing an in-depth knowledge of the entire value network and gives importance to the connection of suppliers with customers and early detection of bottlenecks. Gan et al. [35]. suggested the SC to adopt risk management strategies such as location separation, interdependency, and reliability as a part of the preparation for dealing with unforeseen sudden disruptions. In addition to these, Remko [36] has proposed the research opportunities in SCR considering the post COVID-19 pandemic scenario and focused on the urgency to bridge the gap between industry practices and literature. Modgil et al. [37] has focused on applying artificial intelligence based mechanisms to enhance SCR while identifying the key elements such as visibility as well as sourcing capabilities. Shishodia et al. [38] performed bibliometric and network analysis to critically examine the interconnectedness between the dimensions of SCR. They identified nine key areas for further investigation, which include identifying drivers of SC risks, measuring resilience to enhance SC performance, and emphasizing robustness in SC networks, among others. Haraguchi et al. [39] employed a comparative analysis approach to examine and distinguish between SC disruptions caused by natural disasters and those stemming from the COVID-19 pandemic. They proposed a novel taxonomy of conversion techniques, including production location, production line, storage, distribution channels, and workforce, to address pandemic-related disruptions emphasizing the importance of balancing efficiency and flexibility for SCR. Sunmola et al. [40] centered their study on enhancing SCR through SC visibility. They employed a fuzzy logic approach to prioritize visibility influence factors in SCs, considering two key perspectives: digital technologies and supply chain relationships. The research revealed that the former perspective managers emphasized automation, context awareness, dynamic capability, and information management, while the later perspective managers prioritized SC relationships, management nature, and policy and standards, all contributing to SCR.

### 2.2 Determinants of supply chain resilience

SCs must be resilient in order to stay competitive, and therefore capable of quickly and efficiently recovering from disturbances. Several determinants that facilitate SCR have been studied separately in multidimensional contexts in prior studies. For instance, it has been claimed, certain logistical competencies, when correctly integrated, will result in SCR, which will contribute to long-term survival along with competitive advantage [41]. Further, Ju et al. [42] observed that communication, cooperation, and integration had a significant impact on the development of resilience. Besides, procurement strategies along with sourcing strategies are one of the fundamental factors in the survival and competitiveness of a firm [43, 44]. For instance, having fixed suppliers could exacerbate disruption while having backup suppliers might aid in SCR. Besides, determinants such as visibility and collaboration among the buyers and suppliers are vital to mitigate supplier disruption, avert production and job losses [45, 46]. Collaboration and visibility are powerful strategies for hedging disruption risks, positively impacting suppliers’ recovery rate and buyers’ warning capability. Additionally, visibility enables businesses to pinpoint vulnerable suppliers so that they may prepare contingency plans in case something goes wrong [47]. Needless to say, the necessity of proper information sharing and a holistic collaboration is a must throughout the SC [48].

Therefore, factors or determinants that influence the resilient capabilities of a SC have been identified in different research works. Thorough research of the related works was performed and a total of 25 determinants were identified as the most critical in terms of establishing SCR in companies. They are summarized in **Table 1**.

**Table 1:** Critical determinants of supply chain resilience.

Code	Determinant Name	Brief Explanation	References
D1	Flexible redundancy	It refers to keeping excess capacity by employing extra machines in the production line.	[43]
D2	Emergency suppliers	Emergency suppliers can provide support in supplying quality products within a very short period of time after a disruption occurs.	[49]
D3	Digitalization/modern technology	Organizations should have successful means to adopt and diffuse digital tool and modern technology in their SC.	[50]
D4	Re-routing	A resilient system needs to have the capability to restructure the existing SC and quickly adapt to the changes after a disruption occurs.	[51]
D5	Product flexibility	The products can be designed using standard components which are universally used and readily available globally.	[52]
D6	Managerial coordination and information integration	Management needs to have the experience, authority and knowledge to coordinate and integrate response efforts.	[53]
D7	Shorter lead time	In general, the shorter the supply lead time the faster the disruption in SC can be managed.	[54]
D8	Expansion into e-commerce	The ongoing COVID-19 outbreak has demonstrated the massive shift of customers towards online buying of food and services. In the present scenario, transition of traditional business processes towards e-commerce is critical for developing SCR.	[55]
D9	Responsiveness	This refers to taking emergency decisions and finishing the assigned tasks within steep deadlines under disruption.	[32]
D10	Visibility	This refers to establishing monitoring and event management systems, real time tracking and accountability, throughout the SC.	[56]
D11	Collaboration among stakeholders	This refers to organization wide collaboration among all the stakeholders and also collaboration throughout the SC.	[57]
D12	Geographical segregation	The idea is to have small production areas at different locations which can support the major production area.	[58]
D13	Anticipation and awareness of disruptions	A SC needs to have the culture of analyzing data from past disruptions and find out the underlying patterns, trends and possible intervals of known disruptions.	[59]
D14	Contingent recovery plan	The management of a resilient organization needs to have the ability to run smoothly after disruption.	[60]
D15	Robustness	The production facilities should have the capability to withstand and reduce the extent of damage, after a disruption occurs.	[61]
D16	Level of leanness of production	The level of leanness should be maintained as global competition to reduce SC costs is pushing companies to pursue extreme lean manufacturing practices which is making the SC susceptible to unknown disruptions such as COVID-19 pandemic.	[8]
D17	Level of risk exposure to the outsourcing suppliers	It is imperative to predict and reduce risks resulting from an unplanned disruption such as COVID-19 pandemic.	[62]
D18	Surplus inventory	The existing SC needs to have the ability to meet the uncertainties of demand and supply, by carrying extra stock.	[63]
D19	Re-engineering	This refers to the capability and culture of the SC to correct the errors of the existing system and learn from the deficiencies to handle future disruptions.	[64]
D20	Appropriate location selection	If the area where the facility is going to be located has a history of natural or man-made disasters, then it should not be built there.	[65]
D21	Ease of communication	In order to coordinate response efforts against a disruption, organization wide communication is critical.	[66]
D22	Long-term relationship with suppliers	Long term relationships need to be built up with multiple suppliers before a disruption occurs.	[67]
D23	Degree of offshoring intensity	Outsourcing and offshoring the production process to countries like China, India has enabled the exploitation of cheap labor and low-cost manufacturing, but on the other hand, it increases the vulnerability of SCs to a global pandemic such as COVID-19 pandemic.	[68]
D24	Relationship with competitors	In case of a global disruption, competitor organizations can collaborate with each other on a temporary basis to address the common issues.	[26]
D25	Multimodal transportation	Depending on only one mode of transport can be devastating when a disruption occurs.	[69]

### 2.3 Existing approaches

This section outlines the existing approaches used on SCR in literature. Pramanik et al. [64] in their article identified general determinants of SCR and developed an index for resilient supplier selection using analytic hierarchy process (AHP), technique for order performance by similarity to ideal solution (TOPSIS) and quality function deployment (QFD) under a fuzzy environment. In another article, fuzzy analytic network process (fuzzy ANP) and Grey VIekriterijumsko KOmpromisno Rangiranje (Grey VIKOR) techniques were combined to determine the importance level of the elements effective in resilient SC [70]. Amindoust [71] proposed a resilient-sustainable framework based on the supplier selection indicators using hybrid intelligent method. Davoudabadi et al. [72] proposed a new integrated approach based on interval-valued intuitionistic fuzzy numbers and complex proportional assessment (COPRAS) method for resilient supplier selection. Meanwhile, another research was conducted to develop a united measuring index system to evaluate the resilience of a supplier using fuzzy best worst method (fuzzy BWM) and modular TOPSIS in random environments for group decision-making (GMo-RTOPSIS) method [35]. Shin & Park [61] identified 13 key capabilities as core performance measures of SCR using interpretive structural modeling (ISM) approach. To build a resilient SC during the COVID-19 pandemic, Jain et al. [73] employed a grey-entropy-based approach for assessing the critical success factors and grey-EDAS to evaluate the influence of diverse factors on the resilience of hotel and tourism sector. Sharma and Joshi [74] utilized an integrated method, where stepwise weight assessment ratio analysis (SWARA) for identifying the weightage of factors that affect the selection of digital suppliers and weighted aggregated sum product assessment (WASPAS) for assessing the digital suppliers to explore the best alternative. Towards sustainability and resilience, Mahdiraji et al. [75] provided in-depth insights into the interaction between coordination contracts and challenges of circular economy and eco-innovation focused pharmaceutical SC using SECA approach. In their recent work, Dorfeshan et al. [76] introduced a new model for the integration of SC and project management decisions by incorporating combinative distance-based assessment (CODAS) method and an extended version of BWM. Some of the methods used in this field are summarized in **Table 2**.

**Table 2:** Research contribution and methods used in existing literature.

Authors	Reference	Research Contribution	Used Methods
Pramanik et al., (2017)	[64]	Identifies general determinants of SCR and develops an index for resilient supplier selection	AHP-TOPSIS-QFD under a fuzzy environment
Parkouhi & Ghadikolaei, (2017)	[70]	Determines the importance level of the elements effective in SCR	Fuzzy Analytic Network Process and Grey VIKOR techniques
Amindoust, (2018)	[71]	Proposes a resilient-sustainable framework based on the supplier selection indicators	Hybrid Intelligent Method
Davoudabadi et al., (2019)	[72]	Develops a new approach in resilient supplier selection problem	Interval-valued intuitionistic fuzzy and COPRAS
Shin & Park, (2019)	[61]	Identifies 13 key capabilities as core performance measures of SCR	Interpretive Structural Modelling Approach
Gan et al., (2019)	[35]	Develops a united measuring index system to evaluate the resilience of a supplier	Fuzzy BWM and GMo-RTOPSIS
Jain et al., (2022)	[73]	Explores the critical success factors needed to build a resilient hotel and tourism SC	Grey-Entropy-EDAS
Sharma & Joshi, (2023)	[74]	Examines the factors that influence the selection of digital suppliers and evaluates the criteria for identifying the best supplier that enhances the quality management systems for digital SCs	Integrated SWARA-WASPAS
Mahdiraji et al., (2023)	[75]	Attempts to identify the most significant challenges toward pharmaceutical SC resiliency	Fuzzy version of SECA
Dorfeshan et al., (2023)	[76]	Proposes a new method to determine the resilience score of suppliers	CODAS and BWM

However, it’s important to note that the choice of the most suitable weighting method depends on the specific characteristics of the decision problem, the preferences of the decision-makers, and the quality and availability of data. Fuzzy CRITIC and Fuzzy TOPSIS, like COPRAS, WASPAS, SECA, CODAS, SWARA, MEREC, EDAS, have their own strengths and weaknesses, and the selection of a method should be made based on the particular requirements of the decision-making situation. Additionally, ongoing research and advancements in MCDM methods may lead to refinements and new approaches that further enhance the decision-making process.

### 2.4 Research gaps and contributions

The current body of literature often focuses on individual SCR determinants, such as backup suppliers, visibility, digitalization, flexible allocations and rerouting strategies [77–79], but there is limited research on how these determinants are interrelated across various industries. For example, a study by Shekarian & Mellat Parast [80] primarily considers only four factors as the enhancers of SCR namely flexibility, agility, collaboration and redundancy ignoring the other diverse determinants of SCR. The study also lacks a thorough exploration of the interactions between the said determinants across diverse industries.

While the existing literature frequently emphasizes the significance of SCR determinants, it often fails to offer concrete, quantifiable metrics or measurements for evaluating these determinants and determining their relative importance in setting priorities. Researchers like Alfarsi et al. [81] have explored the strategic importance of SCR, but they lacked a comprehensive quantification method for determining the relative significance of individual determinants. In addition, there is a lack of quantification of the intensity of SCR determinants in terms of their potential variability which can play a critical role in the development of a robust and adaptable SC. Quantifying the variability of SCR determinants aligns with research emphasizing the significance of well-informed supplier selection, the prioritization of high-impact areas, and the implementation of cost-effective decision-making strategies.

To overcome these limitations, the correlation factor of the CRITIC method systematically accounts for the intricate interrelationships between the determinants affecting SCR. To bridge the gap between previous studies, the current work quantifies the priorities of the determinants on the basis of relative importance and provides significant insights into contemporary SC. Moreover, the present work introduces standard deviation to specify the intensity of the determinants across all the alternatives based on its possibility to vary. The study introduces a fuzzy based CRITIC method to ensure completeness and consistency of experts’ opinions. The study also includes an interpretive triple helix framework for establishing SCR. In the realm of SC risk management, it is claimed that resilience has a major role in restoring operations and minimizing risk; but the determinants are not properly investigated in order to achieve resilience.

This research attempts to identify the critical determinants related to SCR. Then it develops an assessment framework for evaluating the determinants and consequently prioritizing them in order of importance. After that, the SCR performances of 10 companies are measured by integrating fuzzy CRITIC and fuzzy TOPSIS methods. Propositions are developed afterward based on the results and finally, an interpretive triple helix framework is incorporated to establish SCR. The major contributions of this work that serve to deal with these research gaps and problems are highlighted in the following:

- Identifying the critical determinants related to SCR through an extensive literature review which is followed by experts’ opinions. This will aid the managers to give a comprehensive knowledge of the determinants that helps to establish a resilient SC.
- Developing an assessment framework for evaluating the determinants as well as prioritizing them in order of importance and measuring the SCR performance of companies by integrating fuzzy CRITIC and fuzzy TOPSIS methods. This prioritization of the determinants is believed to assist the managers in making decisions related to SCR effectively.
- Developing propositions based on the results and applying an interpretive triple helix framework to establish SCR. This will help to combine academia, government, and industry efforts in achieving resilience in the SC.

## 3. Research methodology

MCDM is a technique for making decisions involving the evaluation and selection of alternatives based on multiple criteria or objectives. MCDM approaches can assist decision-makers in considering a variety of criteria, such as economic, social, environmental, and technical factors, and can result in more informed and transparent decisions. The first MCDM method, AHP is a popular approach which was introduced by Thomas Saaty [82–84]. Based on the ideas of AHP, a number of MCDM methods appeared in the literature. For example, ANP, an advanced method of AHP which allows for feedback and dependence among criteria and alternatives [85]; Goal Programming (GP), based on the method of AHP involves setting goals and constraints for each criterion, and then optimizing the objective function subject to these goals and constraints [86]. Besides, fuzzy AHP is an extension of AHP that employs fuzzy logic to make decisions with ambiguous or uncertain data. Likewise, other fuzzy MCDM techniques have drawn interest because they enable the representation of ambiguity and uncertainty inherent in the decision-making process such as fuzzy ANP, fuzzy BWM. Moreover, integrated MCDM methods are also popular because they can help decision-makers to address multiple objectives, multiple criteria, and various forms of data in a more comprehensive and efficient manner. This study has considered an integrated method along with fuzzy inference to get an accurate and robust evaluation of the determinants.

**Fig 1:**
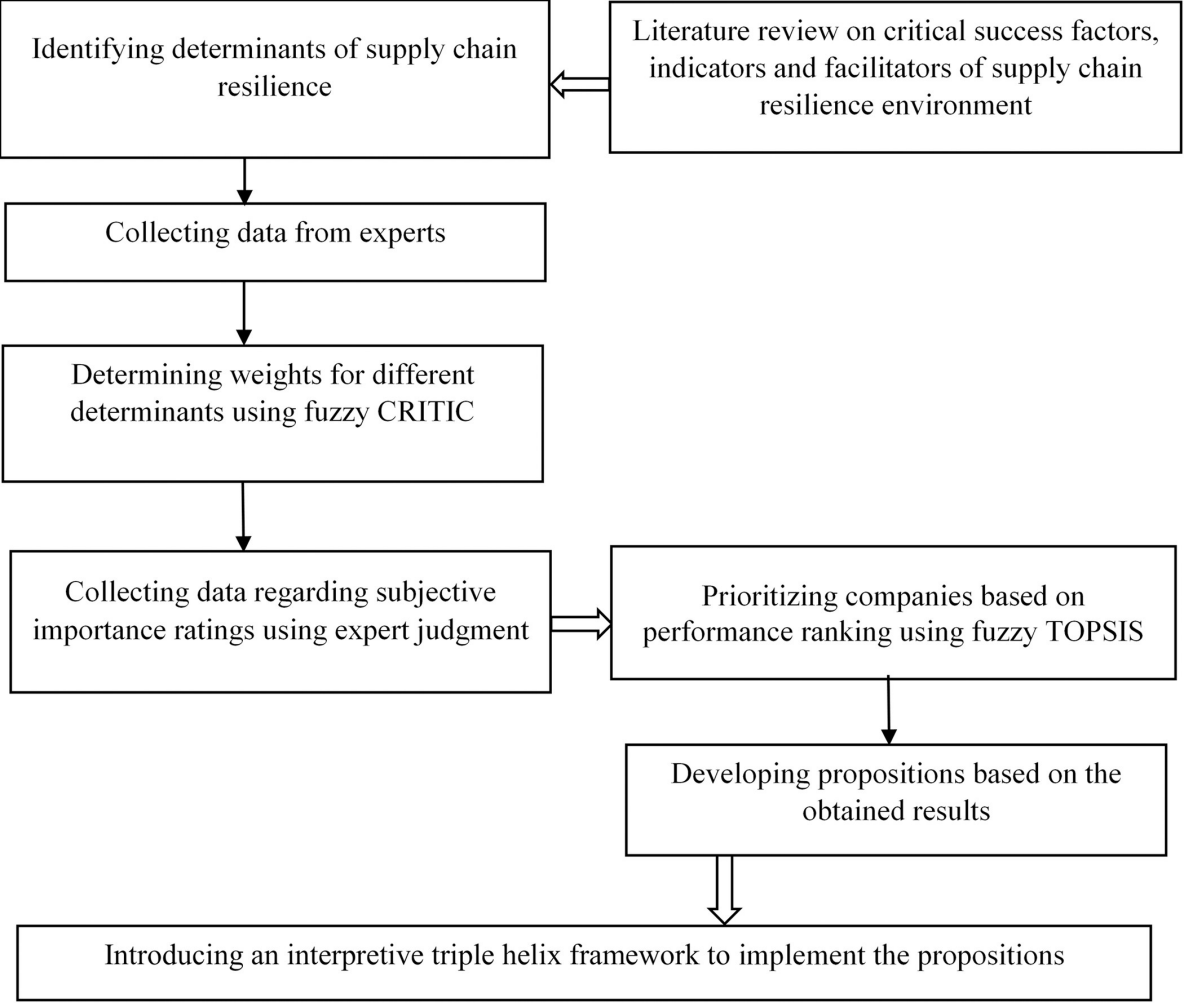
Flow diagram of the research framework.

In this paper, fuzzy CRITIC and fuzzy TOPSIS have been used in the combined form as the solution methodology. The study starts with an extensive literature review to determine the critical determinants related to SCR. Data is collected on companies to determine the objective weights for different determinants using fuzzy CRITIC method. The subjective importance ratings of the determinants are determined using expert judgment. Next, the subjective and the objective weights are combined to determine the relative ranking of the companies using fuzzy TOPSIS method. The companies are prioritized based on performance ranking. Finally, propositions are developed based on the obtained results and an interpretive triple helix framework is introduced to adopt SCR. The proposed research framework is summarized in **Fig 1**.

### 3.1 Fuzzy CRITIC method

Determinants can be used as a parameter in decision-making issues as a knowledge source. The substantial weights of the determinants may represent the amount of information that each of them comprises. The available literature calls these weight of determinants as ‘objective weight’ [19, 87]. The CRITIC is a methodology in the MCDM for the determination of the objective weights of parameters [88]. The weights obtained from this combine both the contrast intensity of each determinant and conflict between the determinants. Contrast intensity of the determinants is considered by the standard deviation and conflict between them is determined by the coefficient of correlation. This method has been expanded here in a fuzzy environment where a fuzzy trapezoidal scale is used for converting the linguistic weights into numerical values and subsequent analysis.

The initial decision matrix is expressed as $Z={\left[z_{ij}\right]}_{m\times n}$ where *z*_*ij*_ is the evaluation value of the i^th^ company according to the j^th^ determinant represented by a trapezoidal fuzzy number $z_{ij}=[b_{ij}^{L},b_{ij}^{ML},b_{ij}^{MU},b_{ij}^{U}]$ where (i = 1, 2,…., m; j = 1, 2,…., n). In order to determine the fuzzy objective weights of determinants, the required steps are mentioned below:

***Step 1*:** The decision matrix $Z={\left[z_{ij}\right]}_{m\times n}$ is normalized into $\tilde{Z}={\left[{\tilde{z}}_{ij}\right]}_{m\times n}$ where ${\tilde{z}}_{ij}=[{\tilde{b}}_{ij}^{L},{\tilde{b}}_{ij}^{ML},{\tilde{b}}_{ij}^{MU},{\tilde{b}}_{ij}^{U}]$ by the following formulas:

For beneficial determinants, we have

$$\left[{\tilde{b}}_{ij}^{L},{\tilde{b}}_{ij}^{ML},{\tilde{b}}_{ij}^{MU},{\tilde{b}}_{ij}^{U}]=[b_{ij}^{L},b_{ij}^{ML},b_{ij}^{MU},b_{ij}^{U}\right]$$ (1)

For non-beneficial determinants, we have

$$\left[{\tilde{b}}_{ij}^{L},{\tilde{b}}_{ij}^{ML},{\tilde{b}}_{ij}^{MU},{\tilde{b}}_{ij}^{U}]=[1-b_{ij}^{U},1-b_{ij}^{MU},1-b_{ij}^{ML},1-b_{ij}^{L}\right]$$ (2)

***Step 2*:** Next, the correlation coefficient *ρ*_*jl*_ between the j^th^ and the l^th^ determinants is calculated by the following formula and the correlation coefficient matrix $\rho ={\left[\rho_{jl}\right]}_{n\times n}$ is obtained:

$$\rho_{jl}=\sum_{i=1}^{m}d({\tilde{z}}_{ij},{\bar{z}}_{j})d({\tilde{z}}_{il},{\bar{z}}_{l})/\sqrt{\sum_{i=1}^{m}d^{2}({\tilde{z}}_{ij},{\bar{z}}_{j})\sum_{i=1}^{m}d^{2}({\tilde{z}}_{il},{\bar{z}}_{l})}$$ (3)

where ${\bar{z}}_{j}$ and ${\bar{z}}_{l}$ are the mean of j^th^ and l^th^ determinants. ${\bar{z}}_{j}$ can be calculated by the following formula. Similarly, ${\bar{z}}_{l}$ can also be obtained.


$${\bar{z}}_{j}=\frac{1}{m}\sum_{i=1}^{m}{\tilde{z}}_{ij}=\frac{1}{m}\sum_{i=1}^{m}{\tilde{b}}_{ij}^{L},\frac{1}{m}\sum_{i=1}^{m}{\tilde{b}}_{ij}^{ML},\frac{1}{m}\sum_{i=1}^{m}{\tilde{b}}_{ij}^{MU},\frac{1}{m}\sum_{i=1}^{m}{\tilde{b}}_{ij}^{U}$$ (4)

***Step 3*:** Then the standard deviation of the j^th^ determinant is calculated by the following formula:

$$s_{j}=\sqrt{\frac{1}{m}\sum_{i=1}^{m}d^{2}({\tilde{z}}_{ij},{\bar{z}}_{j})}$$ (5)

***Step 4*:** The information measures (*I*_*j*_) of the j^th^ determinant is calculated by the following formula:

$$I_{j}=s_{j}\sum_{l=1}^{n}(1-\rho_{jl});j=1,2,\ldots ,n$$ (6)

***Step 5*:** The objective weight vector $w^{o}={\left(w_{1}^{o},w_{2}^{o},\ldots ,w_{n}^{o}\right)}^{T}$ is obtained, where

$$w_{j}^{o}=\frac{I_{j}}{\sum_{j=1}^{n}I_{j}};(j=1,2,\ldots ,n)$$ (7)

### 3.2 Fuzzy CRITIC-TOPSIS combined method

TOPSIS has been a popular approach of determining solutions based on linear programming methods [19, 89]. TOPSIS assumes that there is an ideal and non-ideal solution. The method aims at finding the shortest distance to the positive ideal solution (PIS) and the farthest distance to the negative ideal solution (NIS) [15]. TOPSIS can work with determinant weights or without them. It is a mathematically sound structure. The advantage of the TOPSIS method is that the type of optimization is specified for each determinant (maximize or minimize). In this method, the optimal alternative is chosen based on its proximity to the positive solution while being distant from the negative solution.

In the current study, a set of *m* alternatives represented by *A* and a set of *n* determinants represented by *B* have been assumed. The linguistic decisions are presented as the matrix of outcomes $X={\left[x_{ij}\right]}_{m\times n}$ where *x*_*ij*_ is the performance value of the i^th^ alternative according to the j^th^ determinant represented by a trapezoidal fuzzy number $x_{ij}=[p_{ij}^{L},p_{ij}^{ML},p_{ij}^{MU},p_{ij}^{U}]$ where (i = 1, 2,…., m; j = 1, 2,…., n). The steps of the combined fuzzy CRITIC-TOPSIS method are shown below.

***Step 1*:** The decision matrix is normalized into $\tilde{X}={\left[{\tilde{x}}_{ij}\right]}_{m\times n}$ using the formulas given below where *J* and *J*_1_ represent the beneficial determinants set, and non-beneficial determinants set respectively.

$${\tilde{x}}_{ij}=\left(\frac{p_{ij}^{L}}{d_{j}^{*}},\frac{p_{ij}^{ML}}{d_{j}^{*}},\frac{p_{ij}^{MU}}{d_{j}^{*}},\frac{p_{ij}^{U}}{d_{j}^{*}}\right),j\in J$$ (8)

$${\tilde{x}}_{ij}=\left(\frac{a_{j}^{*}}{p_{ij}^{U}},\frac{a_{j}^{*}}{p_{ij}^{MU}},\frac{a_{j}^{*}}{p_{ij}^{ML}},\frac{a_{j}^{*}}{p_{ij}^{L}}\right),j\in J_{1}$$

where,

$$\begin{array}{c} d_{j}^{*}=\underset{i}{max}p_{ij}^{U},j\in J\\ a_{j}^{*}=\underset{i}{min}p_{ij}^{L},j\in J_{1} \end{array}$$ (9)

***Step 2*:** The subjective determinants weights’ matrix $w^{s}={\left[w_{j}^{s}\right]}_{n\times 1}$ is constructed using the following equations.

$$w_{j}^{s}=\frac{1}{r}\begin{array}{c} r\\ ⊕\\ p=1 \end{array}w_{jp}^{s}$$ (10)

where,

$w_{jp}^{s}$ represents the subjective weight of determinant *B*_*j*_ assigned by the *p*^th^ decision-maker where (*p* = 1, 2,…., r).

***Step 3*:** In this step the aggregated weights are determined by combining the subjective weights and the objective weights, using the following equation.

$$w_{j}=\rho \cdot w_{j}^{s}⊕(1-\rho )\cdot w_{j}^{o}$$ (11)

where,

w_*j*_ = aggregated weights

$w_{j}^{s}$ = subjective weights

$w_{j}^{o}$ = objective weights

*ρ* = weight factor

***Step 4*:** Then the matrix of weighted normalized fuzzy decision is formulated using the following equations.

$$\begin{array}{l} T={{[\bar{t}}_{ij}]}_{m\times n}\\ {\bar{t}}_{ij}=w_{j}⊗{\tilde{x}}_{ij} \end{array}$$ (12)

where,

*T =* Weighted Normalized fuzzy decision matrix

${\bar{t}}_{ij}$ = Weighted normalized fuzzy value of j^th^ determinant of i^th^ alternative

***Step 5*:** The fuzzy negative ideal solution (FNIS) and fuzzy positive ideal solution (FPIS) are determined using the following equations.

$$A^{+}=(v_{1}^{+},v_{2}^{+},\ldots ,v_{n}^{+})$$ (13)

$$A^{-}=(v_{1}^{-},v_{2}^{-}{,\ldots ,v}_{n}^{-})$$

$$\begin{array}{l} where,\\ v_{j}^{+}=\{\begin{array}{cc} \underset{i}{max}\;p_{ij}^{U} & \mathrm{if}\;j\in J\\ \underset{i}{min}\;p_{ij}^{L} & \mathrm{if}\;j\in J_{1} \end{array}\\ v_{j}^{-}=\{\begin{array}{cc} \underset{i}{min}\;p_{ij}^{L} & \mathrm{if}\;j\in J\\ \underset{i}{max}\;p_{ij}^{U} & \mathrm{if}\;j\in J_{1} \end{array} \end{array}$$ (14)

where,

*A*^+^ *=* The set of fuzzy positive ideal solution (FPIS)

$v_{j}^{+}$ *=* Fuzzy positive ideal solution of j^th^ determinant

*A*^−^ *=* The set of fuzzy negative ideal solution (FNIS)

$v_{j}^{-}$ *=* Fuzzy negative ideal solution of j^th^ determinant

***Step 6*:** In this step the distances of each company from FNIS and FPIS are determined using the following equations.

$$\begin{array}{c} o_{i}^{+}=\sum_{j=1}^{n}o\left({\bar{t}}_{ij}\cdot v_{j}^{+}\right)\\ o_{i}^{-}=\sum_{j=1}^{n}o\left({\bar{t}}_{ij}\cdot v_{j}^{-}\right) \end{array}$$ (15)

where,

$o_{i}^{+}$ *=* Distance of i^th^ alternative from FPIS

$o_{i}^{-}$ = Distance of i^th^ alternative from FNIS

***Step 7*:** Finally, the relative closeness of each company which were the alternatives is calculated and then according to descending order of these values using the following equations they have been ranked.

$$C_{i}=\frac{o_{i}^{-}}{o_{i}^{-}+o_{i}^{+}}$$ (16)

where,

*C*_*i*_ *=* Relative closeness of the i^th^ alternative

**Fig 2:**
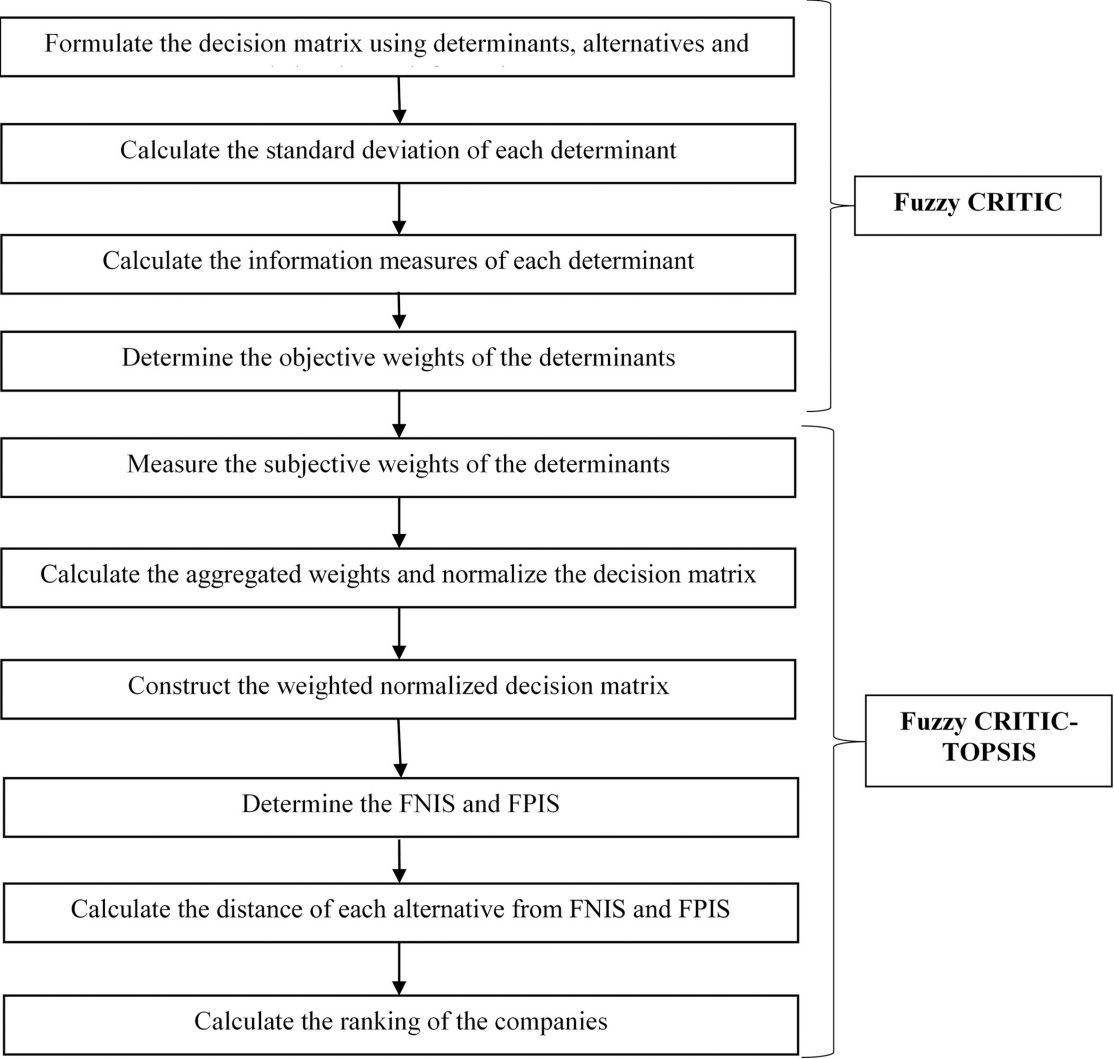
Flow diagram of the fuzzy CRITIC-TOPSIS approach.

The combined fuzzy CRITIC-TOPSIS approach is illustrated in **Fig 2**.

## 4. Result and discussion

### 4.1 Data collection

Based on the literature review and the contribution of the authors of this paper, 25 determinants of SCR were identified as shown in **Table 1**. Then a questionnaire **(see S1 File)** was developed and consequently distributed among 10 experts working in the relevant field. The profile of the experts of this study has been mentioned in **Table A** in **S2 File**. In this study, 10 companies (C1, C2, C3, C4, C5, C6, C7, C8, C9 and C10) were selected to perform the analysis. The profile of the companies can be found in **Table B** in **S2 File**. The companies, representing diverse industries like textile, paint, retail, consumer goods, fashion, conglomerates, food, telecommunication, automotive, and confectionery, provide a diverse view of SC disruption risks due to their global reach and industry-specific challenges. Textile and paint companies like C1 and C2 respectively are subject to raw material shortages and transportation issues which make them susceptible to sudden disruptions in SC. C3, a global retailer, faces risks in the form of trade dispute and can affect millions of customers and suppliers in the event of a natural disaster or a global pandemic. Consumer goods giant C4 relies on complex distribution networks and sourcing. Fashion company C5 faces risks in its globalized SCs making them vulnerable to disruptions in logistics, labor, and raw material supply. Conglomerates like C6 with diverse interests in sectors such as automotive, energy, and retail have multiple industry-specific vulnerabilities. Given its susceptibility to SC disruptions in the form of port congestion, supplier reliability, communication and coordination gaps, labor strikes and other logistical challenges, a resilient strategy can ensure business stability and minimize the impact on its diverse operations. Food manufacturer C7 relies on a smooth ingredient supply. On the other hand, a telecommunications company like C8 faces potential disruptions including component shortages, geopolitical tensions, cybersecurity threats, and supplier dependencies, emphasizing the importance of SCR measures for uninterrupted operations. Automotive parts manufacturer C9 is vital for the automotive industry’s SC and is sensitive to SC disruptions due to the reliance on just-in-time manufacturing and complex global supply networks. Confectionery company C10 depends on global ingredient movement making them vulnerable to SC interruptions. Analyzing these companies helps understand various SC vulnerabilities, from sourcing to logistics, geopolitical influences, and industry-specific challenges.

### 4.2 Result analysis

A seven point fuzzy scale was used where the linguistic scales for rating used in this study are presented in **Table C** in **S2 File**. The importance weight of determinants as assessed by experts can be found in **Table D** in **S2 File**. The study has collected data from 10 individual companies. The ratings of the companies as assessed by the corresponding company employees can be found in **Table E** in **S2 File**. Using the fuzzy linguistic scales, a decision matrix was formulated which can be found in **Table F** in **S2 File**. Fuzzy CRITIC method was used to determine the objective weights which can be found in **Table G** in **S2 File**. Subjective weights and defuzzified subjective weights are also provided in the same table. The subjective and the objective weights were combined to form aggregated weights which can be found in **Table H** in **S2 File**. Standard deviation was determined, and the determinants were ranked based on the standard deviation which can be found in **Table I** in **S2 File**. This ranking specifies the intensity of the determinants on their possibility to vary. The decision matrix was normalized and provided in **Table J** in **S2 File**. Then the normalized decision matrix was multiplied by the aggregated weights to get the weighted normalized decision matrix which is provided in **Table K** in **S2 File**. The fuzzy positive ideal solutions (FPIS) and distance of each company from FPIS were determined which can be found in **Table L** in **S2 File**. The fuzzy negative ideal solutions (FNIS) and distance of each company from FNIS is provided in **Table M** in **S2 File**. Finally, the relative closeness of each company was determined and ranked which can be found in **Table N** in **S2 File**. This ranking specifies the degree to which each company exercises SCR practices.

### 4.3 Findings of the study

There are three rankings in our study. The first ranking is based on the importance of determinants for establishing SCR which can be seen from **Table 3**. In this ranking ‘Responsiveness (D9)’ was found to be the most important determinant followed by ‘Managerial coordination and information integration (D6)’, and ‘Emergency suppliers (D2)’. ‘Surplus inventory (D18)’ was found to be the least important determinant in order to establish a resilient SC.

**Table 3:** Prioritization of determinants based on importance.

Determinant Code	Rank
D9	1
D6	2
D2	3
D8	4
D22	5
D14	6
D11	7
D21	8
D10	9
D7	10
D17	11
D19	12
D3	13
D15	14
D13	15
D4	16
D16	17
D5	18
D25	19
D23	20
D20	21
D12	22
D1	23
D24	24
D18	25

The second ranking is based on the performance of companies regarding SCR practices. Company C8 was found to be the best practitioner of SCR practices and company C10 was found to be the worst practitioner, as shown in **Table 4**.

**Table 4:** Relative ranking of the companies.

Company Code	Rank
C8	1
C3	2
C2	3
C9	4
C1	5
C4	6
C6	7
C5	8
C7	9
C10	10

The third ranking is based on the intensity of the determinants regarding their possibility to vary across companies. The measure of ‘Appropriate location selection (D20)’ was found to vary the most across the companies and ‘Emergency suppliers (D2)’ varied the least. The ranking is given below in **Table 5**.

**Table 5:** Intensity of the determinants based on its possibility to vary across companies.

Determinant Code	Rank
D20	1
D8	2
D19	3
D24	4
D16	5
D12	6
D21	7
D13	8
D4	9
D17	10
D1	11
D25	12
D10	13
D11	14
D18	15
D23	16
D9	17
D15	18
D5	19
D14	20
D3	21
D6	22
D7	23
D22	24
D2	25

### 4.4 Triple helix framework to establish supply chain resilience

As per research question-3 (***RQ3***), the study proposes a cross-disciplinary approaches, i.e., a triple helix framework where three independent actors namely government, industry and academia can effectively collaborate to establish a resilient SC.

#### 4.4.1 Academia-Industry interaction

Industries must collaborate with universities by providing real time data and sponsoring fund for research and development (R & D) focusing on resilient SC practices [90, 91]. Academia then can use this data for subsequent analysis. This will generate cutting edge knowledge and technological innovations to better handle future disruptions [92]. This will not only further the study in this field but also establish the significance of resilient practices in the contemporary SC environment. Academia can also impart knowledge-based training in the area of data science, forecasting, predictive analytics to the company professionals which will help to develop skilled human capital [93]. Thus, academia and industries can build partnerships and effective cooperation networks for R & D to achieve resilience in the SC.

#### 4.4.2 Government-Industry interaction

The government needs to generate policies to better handle sudden SC disruptions in the future [94]. These policies then need to be implemented strongly in the industries. The industries can be given incentives such as tax rebates on complying with the regulations [95, 96]. In this way, an infrastructure based on policy and compliance needs to be established between the government and industry. In this collaboration between the government and the industry, the government holds financial capital and the industry holds productive capital. Effective cooperation between these two capitals can help to achieve resilience in the SC [97].

#### 4.4.3 Academia-Government interaction

The government can fund research activities collaborating with academia to develop innovative strategies and technologies. New knowledge and technological innovations help industries sustain in the face of unforeseen disruptions which in turn keeps the industries running and jobs secure [98]. The government needs to patronize the generation of knowledge and technological innovations through monetary support. The government gets the money back in the form of taxes and creates stable jobs. Governments in this regard can influence academic curriculum to focus on the prediction and handling of sudden disruptions and sponsor research on relevant issues.

#### 4.4.4 Academia-Government-Industry interaction

The three-way collaboration among the most significant actors in the SC paves the way for developing a dynamic, interpretive and continuous process of learning. This makes way for the generation of innovative strategies and technological breakthroughs for rapid response to SC disruptions. As the academia generates resources on better handling of sudden disruptions in the SC, the government can raise awareness on the sponsored research findings through media and technology and can implement the application of the findings by framing mandatory policies for the industries. With the help of academia, the government can implement training programs for the company professionals to better prepare them for future disruptions in SC.

**Fig 3:**
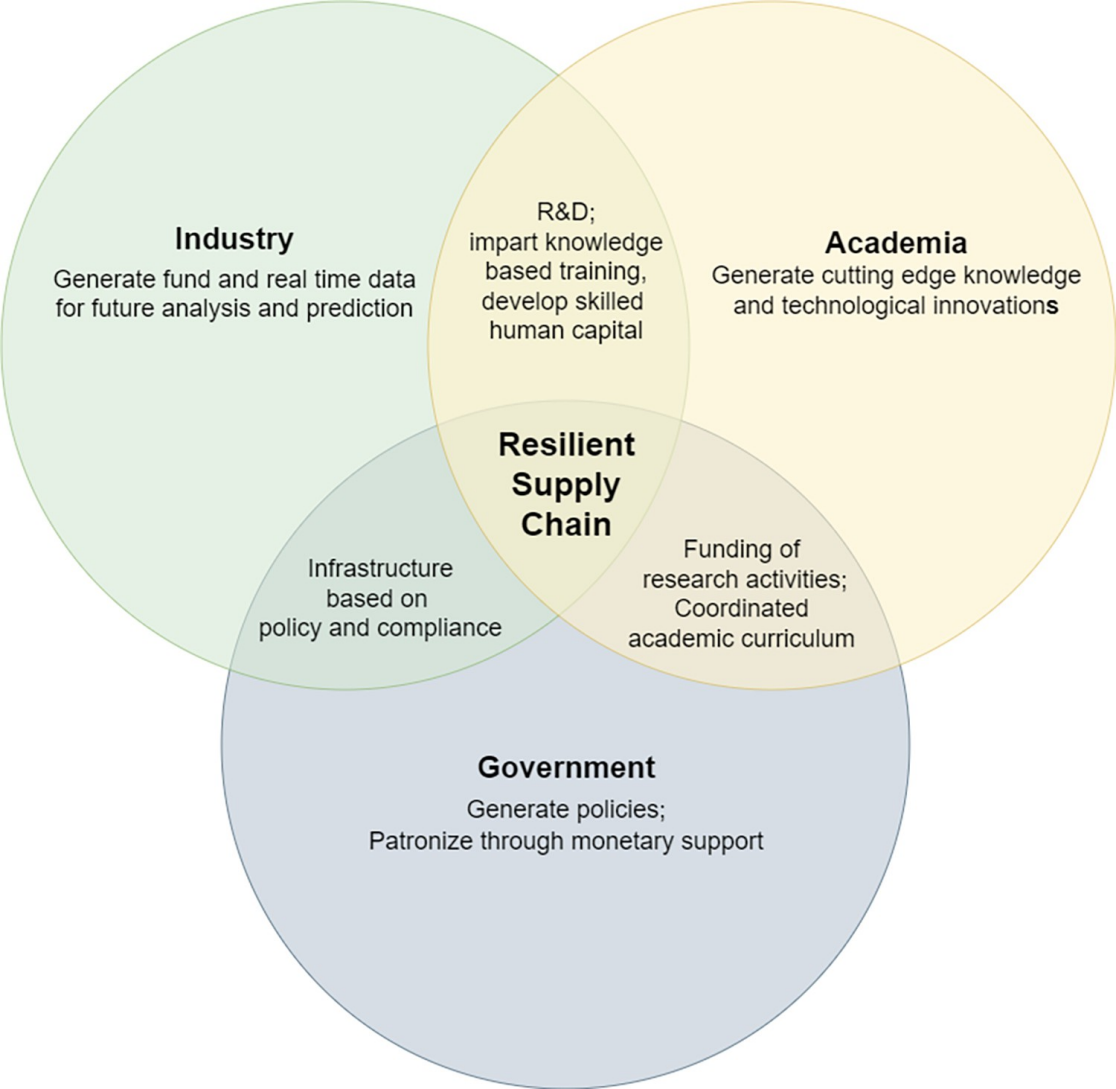
Triple helix framework to establish supply chain resilience.

**Fig 3** shows a visual representation of our proposed triple helix framework.

## 5. Contribution and implications

### 5.1 Theoretical contribution

This research has four major theoretical implications-

First, this study represents a meaningful advancement in the exploration of SCR by incorporating diverse perspectives within both local and global contexts, fostering a robust research model poised for future investigations. While extant literature often dissects specific factors influencing SCR, such as alternative suppliers, SC visibility, digitization, flexible allocation, and rerouting tactics, a notable gap exists in understanding the interconnectedness of these drivers across varied industries. This study addresses this void by elucidating the most crucial determinants of resilience across diverse industries, ranging from fast-moving consumer goods (FMCG) to telecommunications while incorporating the CRITIC method which systematically incorporates the correlation factor, accounting for intricate interconnectedness among the determinants of SCR.

Second, this research proposes an assessment framework using fuzzy based MCDM tools with triple helix framework to evaluate the SCR performance. The proposed framework can act as a robust tool for managing uncertainty and vagueness in decision problems characterized by imprecise information, with Fuzzy CRITIC providing a nuanced reflection of the SC environment, particularly in assessing the importance of diverse criteria. This framework not only enables companies to measure their resilience performance but also facilitates performance comparisons among different companies. Additionally, the introduction of standard deviation in this study, adds to the current body of literature. by making it possible to assess the intensity of these determinants across alternatives, offering insights into their variability.

Third, this research contributes to the existing literature on SCR by linking three independent actors namely government, industry and academia in the form of a triple helix framework which will help the researchers to develop a more resilient and reliable framework for SCR in the future.

Fourth, the implementation of the suggested framework has found ‘Responsiveness (D9)’, ‘Managerial coordination and information integration (D6)’, and ‘Emergency suppliers (D2)’ to be the topmost important determinants for establishing SCR. This will enhance the existing body of literature by offering a perspective for future researchers to explore these determinants of SCR in subsequent investigations.

This study makes a unique contribution to the existing literature in the form of the theme, unique perspective in enriching SCR comprehension, identifying and prioritizing determinants, thereby advancing a more comprehensive theoretical framework for evaluating and fortifying SCR in the face of disruptions, promoting a resilient culture across industries.

### 5.2 Managerial implications

This research will aid the decision makers and policymakers to understand the significant/key determinants relevant in their SC to facilitate the SCR culture in industries. Understanding the determinants of SCR can help the managers assess the vulnerabilities and strengths of their SC and by focusing on relevant metrics, they will be able to determine the effectiveness of their existing resilience strategies and practices. It can support a culture of continuous improvement, where feedback loops, post-event reviews, and learning from past disruptions can lead to adaptive and resilient SC.

This study identifies ‘Responsiveness (D9)’ as vital determinant that should be given highest priority to achieve SCR. Responsiveness involves addressing customer complaints, concerns, and inquiries promptly. Organizations that prioritize and implement responsive strategies are better positioned to adapt to changing circumstances, seize opportunities, and build long-term resilience [99]. Managers need to ensure open lines of communication (D21), collaboration (D11), and contingency planning (D14) with stakeholders to ensure a reliable and responsive SC. The COVID-19 pandemic exemplifies the significance of responsiveness in building a resilient SC [100, 101]. Companies that were agile, adaptable, and quick to respond to changing circumstances were better positioned to overcome disruptions, meet customer needs, and maintain business continuity [99, 102]. Responsiveness, in this context, was not just a theoretical concept but a practical necessity for surviving and thriving during a crisis.

The study also reveals that the industries should focus on ‘Managerial coordination and information integration (D6)’ activities. Managerial coordination among SC partners is critical for building resilience [103, 104]. Effective coordination among SC partners involves aligned goals, information sharing, and collaborative decision-making which can significantly increase SCR [105]. On the other hand, information integration through shared IT systems, data analytics, and communication platforms can enable real-time visibility and transparency across the SC, aiding in faster response and recovery.

Another finding of the study reveals that the industrial managers should invest more in long-term relationships with emergency suppliers i.e., D2, D22. It can provide a backup source of supply during disruptions or crises when the primary suppliers are unable to deliver. This helps ensure a continuous flow of materials or products, minimizing disruptions and downtime in the SC [106, 107]. ‘Expansion into E-commerce (D8)’ has been found to be crucial for strengthening SCR as it allows for greater visibility, real-time data-driven decision-making, and diversified sales channels reducing vulnerability to disruptions. Managers in this regard should develop and invest in e-commerce capabilities, digital platforms and robust data analytics while fostering collaboration between IT and operations teams to enhance SCR.

The findings of the study indicate that the managers should increase procurement from multiple suppliers and establish collaboration and visibility among all the stakeholders i.e., D10, D11. Procuring from multiple suppliers reduces reliance on a single source, mitigating the risk of disruptions caused by issues such as supplier bankruptcies, natural disasters, or geopolitical conflicts [108]. Industrial managers should have a thorough knowledge of the risk exposure to the outsourcing suppliers before making any offshoring decision (D23). By understanding the risk exposure of potential outsourcing suppliers, companies can assess the risks associated with offshoring decisions [1]. This can include evaluating risks such as geopolitical risks, transportation disruptions, currency fluctuations, labor strikes, or supplier bankruptcy. Another finding is that the degree of leanness of the production (D16) should not exceed the degree of risks associated with it. Lean operations typically emphasize minimizing inventory levels to reduce waste and increase efficiency. However, having little or no buffer inventory can leave the SC vulnerable to disruptions, such as unexpected demand fluctuations, transportation delays, or supplier disruptions [109].

## 6. Conclusion, limitations and scope for future research

SCR is a pressing issue, especially realized during the disruption caused by the ongoing COVID-19 pandemic situation. This study presents the key determinants related to SCR and discusses the study implication based on the ‘triple helix framework’. This paper demonstrates determinants and propositions to build SCR which minimize the impacts of disruption risks faced by SCs. By focusing on the crucial determinants, the resilient SC should be able foresee unexpected disruptive events, sustain operation, recover quickly from disruptions and ultimately gain competitive advantage. This research prioritizes the determinants such as ‘responsiveness’, ‘managerial coordination and information integration’, ‘emergency suppliers’, and ‘expansion into e-commerce’. These findings corroborate the significance of these determinants in enhancing SCR, aligning with developed proposition using triple helix framework. This work contributes by incorporating a correlation factor that accounts for the inter-determinant correlations and overcomes previous limitations by introducing standard deviation to specify the intensity of the determinants across all the companies based on its possibility to vary. The revelation of these concerns has prompted suggestions for better risk mitigation via the creation of SCR. The significant advantage of the proposed CRITIC method lies in its capacity to estimate criteria weights by accounting for randomness and variations in the performance ratings of the alternatives, employing well-developed algorithms. In contrast, TOPSIS, as another widely-used method, determines the Euclidean distances of each alternative from both the ideal and anti-ideal solutions. These approaches showcase different strengths in the realm of multi-criteria decision-making, with CRITIC focusing on robust weight estimation and TOPSIS emphasizing geometric distance calculations. One of the limitations of the study is the fact that the fuzzy weights of determinants were determined by experts’ judgments. This can vary from expert to expert which subsequently affects the evaluation of the determinants and performance analysis of the companies. Fuzzy CRITIC’s complexity, data-intensive requirements, and subjectivity, along with Fuzzy TOPSIS’s sensitivity to fuzzy set parameters and computational demands, present notable challenges for their application in decision-making contexts. However, this can be overcome by collecting data using multiple rounds of questionnaires followed by subsequent analysis until a desired stability in the result is obtained. For this purpose, we recommend using the Delphi method. We intend to extend our study using the Delphi method in the future. As future research scopes, the results of the current study can be verified and compared by the application of other newly developed fuzzy MCDM tools.

## Supporting information

S1 File Questionnaire. (DOCX)

S2 File Supplementary information file. (DOCX)
