# Supplementary material for: Key determinants to supply chain resilience to face pandemic disruption: An interpretive triple helix framework
Source: PLoS One. 2024 May 1;19(5):e0299778. doi: 10.1371/journal.pone.0299778 (PMC11062547; doi:10.1371/journal.pone.0299778)
Supplement: S1 File — (DOCX) [file pone.0299778.s001.docx]

**S1 File. Questionnaire**

1. In which industry sector are you working?

2. For how many years have you worked in the respective sector?

1-3 years

4-9 years

More than 9 years

3. What is your position in the company?

4. Establishment year of the company-

Before 2000

2001-2010

After 2010

5. Status of the company-

Fully Export Oriented

Partially Export Oriented

Fully Local Oriented

6. Annual turnover/ revenue of the company-

More than USD 500 million

Less than USD 500 million

7. Which International Organization for Standardization (ISO) certification does your company have?

ISO 9000

ISO 14000

**Question 1:** How important is the determinant for attaining supply chain resilience?

| **Determinant** | **Very Low/ Very Poor** | **Low/ Poor** | **Medium Low/ Medium Poor** | **Medium** | **Medium High/ Medium Good** | **High/ Good** | **Very High/ Very Good** |
| --- | --- | --- | --- | --- | --- | --- | --- |
| Flexible redundancy |  |  |  |  |  |  |  |
| Emergency suppliers |  |  |  |  |  |  |  |
| Digitalization/modern technology |  |  |  |  |  |  |  |
| Re-routing |  |  |  |  |  |  |  |
| Product flexibility |  |  |  |  |  |  |  |
| Managerial coordination and information integration |  |  |  |  |  |  |  |
| Shorter lead time |  |  |  |  |  |  |  |
| Expansion into E-commerce |  |  |  |  |  |  |  |
| Responsiveness |  |  |  |  |  |  |  |
| Visibility |  |  |  |  |  |  |  |
| Collaboration among stakeholders |  |  |  |  |  |  |  |
| Geographical segregation |  |  |  |  |  |  |  |
| Anticipation and awareness of disruptions |  |  |  |  |  |  |  |
| Contingent recovery plan |  |  |  |  |  |  |  |
| Robustness |  |  |  |  |  |  |  |
| Level of leanness of production |  |  |  |  |  |  |  |
| Level of risk exposure to the outsourcing suppliers |  |  |  |  |  |  |  |
| Surplus inventory |  |  |  |  |  |  |  |
| Re-engineering |  |  |  |  |  |  |  |
| Appropriate location selection |  |  |  |  |  |  |  |
| Ease of communication |  |  |  |  |  |  |  |
| Long-term relationship with suppliers |  |  |  |  |  |  |  |
| Degree of offshoring intensity |  |  |  |  |  |  |  |
| Relationship with competitors |  |  |  |  |  |  |  |
| Multimodal transportation |  |  |  |  |  |  |  |

**Question 2:** How will you rate the performance of your company on this factor?

| **Determinant** | **Very Low/ Very Poor** | **Low/ Poor** | **Medium Low/ Medium Poor** | **Medium** | **Medium High/ Medium Good** | **High/ Good** | **Very High/ Very Good** |
| --- | --- | --- | --- | --- | --- | --- | --- |
| Flexible redundancy |  |  |  |  |  |  |  |
| Emergency suppliers |  |  |  |  |  |  |  |
| Digitalization/modern technology |  |  |  |  |  |  |  |
| Re-routing |  |  |  |  |  |  |  |
| Product flexibility |  |  |  |  |  |  |  |
| Managerial coordination and information integration |  |  |  |  |  |  |  |
| Shorter lead time |  |  |  |  |  |  |  |
| Expansion into E-commerce |  |  |  |  |  |  |  |
| Responsiveness |  |  |  |  |  |  |  |
| Visibility |  |  |  |  |  |  |  |
| Collaboration among stakeholders |  |  |  |  |  |  |  |
| Geographical segregation |  |  |  |  |  |  |  |
| Anticipation and awareness of disruptions |  |  |  |  |  |  |  |
| Contingent recovery plan |  |  |  |  |  |  |  |
| Robustness |  |  |  |  |  |  |  |
| Level of leanness of production |  |  |  |  |  |  |  |
| Level of risk exposure to the outsourcing suppliers |  |  |  |  |  |  |  |
| Surplus inventory |  |  |  |  |  |  |  |
| Re-engineering |  |  |  |  |  |  |  |
| Appropriate location selection |  |  |  |  |  |  |  |
| Ease of communication |  |  |  |  |  |  |  |
| Long-term relationship with suppliers |  |  |  |  |  |  |  |
| Degree of offshoring intensity |  |  |  |  |  |  |  |
| Relationship with competitors |  |  |  |  |  |  |  |
| Multimodal transportation |  |  |  |  |  |  |  |
