# Supplementary material for: Key determinants to supply chain resilience to face pandemic disruption: An interpretive triple helix framework
Source: PLoS One. 2024 May 1;19(5):e0299778. doi: 10.1371/journal.pone.0299778 (PMC11062547; doi:10.1371/journal.pone.0299778)
Supplement: S2 File — (DOCX) [file pone.0299778.s002.docx]

**S2 File. Supplementary information file**

**Table A. Profile of the experts**

| **Expert Code** | **Position** | **Years of experience** | **Area** |
| --- | --- | --- | --- |
| **E1** | Manufacturing Director | 18 years | Textile industry |
| **E2** | Manager, Planning and Purchase | 18 years | Paint and construction chemicals industry |
| **E3** | Sourcing Analyst | 17 years | Customer fulfilment |
| **E4** | Senior Manager, Procurement | 16 years | Health, hygiene based industry |
| **E5** | Manager, Manufacturing Excellence | 13 years | Fashion industry |
| **E6** | Head of Supply Chain | 12 years | Automotive industry |
| **E7** | Continuous Improvement TPM Manager, Corporate Division | 9 years | Food industry |
| **E8** | Sourcing Manager | 7 years | Telecommunication industry |
| **E9** | Engineering Specialist | 7 years | Manufacturing |
| **E10** | SCM Executive | 5 years | Sugar candy industry |

**Table B. Profile of the companies**

| **Company Code** | **Number of Employee** | **Product Type** | **Annual Turnover** | **Export Orientation** |
| --- | --- | --- | --- | --- |
| **C1** | 800 | Textile fabric, thread, adhesives | Less than USD 500 million | Fully export oriented |
| **C2** | 1000 | Paint and construction chemicals | More than USD 500 million | Partially export oriented |
| **C3** | 6300 | Furniture | More than USD 500 million | Fully export oriented |
| **C4** | 230 | Health and hygiene products | More than USD 500 million | Partially export oriented |
| **C5** | 950 | Fashion wear | More than USD 500 million | Fully export oriented |
| **C6** | 3500 | Battery, IPS, UPS, automotive products | Less than USD 500 million | Partially export oriented |
| **C7** | 2500 | Food | More than USD 500 million | Partially export oriented |
| **C8** | 1900 | Telecommunication | More than USD 500 million | Fully export oriented |
| **C9** | 307 | Automotive | More than USD 500 million | Partially export oriented |
| **C10** | 1200 | Sugar candy | Less than USD 500 million | Fully local oriented |

**Table C.** **Linguistic scales for importance and ratings.**

| **Linguistic Scale** | **Fuzzy Scale** | **Linguistic Scale for Rating** |
| --- | --- | --- |
| Very Low (VL) | (0, 0, 0.1, 0.2) | Very Poor (VP) |
| Low (L) | (0.1, 0.2, 0.2, 0.3) | Poor (P) |
| Medium Low (ML) | (0.2, 0.3, 0.4, 0.5) | Medium Poor (MP) |
| Medium (M) | (0.4, 0.5, 0.5, 0.6) | Medium (M) |
| Medium High (MH) | (0.5, 0.6, 0.7, 0.8) | Medium Good (MG) |
| High (H) | (0.7, 0.8, 0.8, 0.9) | Good (G) |
| Very High (VH) | (0.8, 0.9, 1, 1) | Very Good (VG) |

**Table D.** **Importance weight of determinants as assessed by experts.**

| **Code** | **E1** | **E2** | **E3** | **E4** | **E5** | **E6** | **E7** | **E8** | **E9** | **E10** |
| --- | --- | --- | --- | --- | --- | --- | --- | --- | --- | --- |
| **D1** | MH | MH | L | H | M | VH | M | M | L | MH |
| **D2** | H | VH | H | VH | MH | VH | H | H | VH | VH |
| **D3** | VH | VH | H | VH | ML | MH | M | MH | VH | H |
| **D4** | VH | MH | H | H | MH | H | ML | H | VH | ML |
| **D5** | ML | MH | VH | MH | MH | H | H | VH | MH | M |
| **D6** | VH | VH | H | H | H | H | VH | VH | VH | H |
| **D7** | MH | H | VH | VH | M | VH | ML | VH | VH | H |
| **D8** | H | VH | VH | VH | H | H | H | VH | VH | ML |
| **D9** | VH | VH | VH | H | VH | VH | H | VH | VH | MH |
| **D10** | VH | VH | H | H | H | VH | H | H | MH | ML |
| **D11** | H | H | H | H | MH | H | M | VH | H | VH |
| **D12** | ML | MH | H | ML | MH | H | L | H | MH | VH |
| **D13** | L | MH | H | H | H | H | MH | VH | VH | MH |
| **D14** | MH | VH | VH | MH | H | VH | H | H | VH | M |
| **D15** | ML | MH | VH | H | M | VH | MH | VH | VH | H |
| **D16** | H | H | VH | VH | L | VH | M | VH | H | L |
| **D17** | H | VH | H | MH | ML | H | H | VH | VH | MH |
| **D18** | MH | ML | H | ML | ML | H | ML | L | L | VH |
| **D19** | H | H | VH | M | MH | VH | MH | H | VH | M |
| **D20** | VH | M | VH | H | L | VH | L | H | VH | L |
| **D21** | VH | VH | VH | H | L | VH | VH | H | VH | L |
| **D22** | H | VH | VH | VH | M | VH | H | M | VH | MH |
| **D23** | VH | H | H | M | M | MH | MH | M | MH | M |
| **D24** | H | M | L | H | MH | MH | L | M | VH | L |
| **D25** | VH | H | VH | VH | M | MH | M | H | MH | L |

**Table E. Ratings of the companies as assessed by the corresponding company employees.**

| **Code** | **C1** | **C2** | **C3** | **C4** | **C5** | **C6** | **C7** | **C8** | **C9** | **C10** |
| --- | --- | --- | --- | --- | --- | --- | --- | --- | --- | --- |
| **D1** | MG | G | P | G | M | MG | MG | VG | G | M |
| **D2** | G | G | G | G | MG | MG | MG | VG | VG | MG |
| **D3** | G | VG | G | G | MP | MG | MG | G | MG | MG |
| **D4** | VG | G | G | VG | MG | G | MP | MG | M | MP |
| **D5** | MP | G | G | M | MG | MG | MP | MG | M | MG |
| **D6** | VG | G | G | M | G | MG | MG | VG | VG | MG |
| **D7** | MG | G | G | MG | M | MG | MG | VG | M | MG |
| **D8** | MP | G | VG | M | G | M | MP | VG | G | P |
| **D9** | VG | G | G | M | VG | MG | G | VG | MG | M |
| **D10** | G | VG | MG | G | G | M | MP | G | M | M |
| **D11** | VG | MG | MG | M | MG | MG | MP | VG | MG | M |
| **D12** | P | M | G | MP | MG | M | MP | VG | MG | MG |
| **D13** | P | MG | G | G | G | M | M | VG | G | G |
| **D14** | MG | G | VG | MG | G | MG | MP | MG | MG | M |
| **D15** | MP | MG | G | M | MG | M | M | G | MG | MP |
| **D16** | MG | MG | G | G | P | G | M | VG | MG | MP |
| **D17** | G | VG | G | MG | MP | M | MG | VG | G | MP |
| **D18** | MG | MP | MG | MP | M | MG | M | VG | MG | MG |
| **D19** | M | G | G | M | M | MG | MP | VG | MG | VP |
| **D20** | G | M | VG | G | P | G | P | VG | VG | MP |
| **D21** | VG | VG | VG | G | P | MG | G | G | VG | G |
| **D22** | G | VG | VG | G | G | M | MG | G | VG | G |
| **D23** | MG | G | VG | M | G | MG | G | G | MG | MP |
| **D24** | P | MP | P | M | VG | MG | P | M | M | MP |
| **D25** | VG | G | VG | VG | M | MG | M | G | MG | MP |

**Table F. Decision matrix.**

| **Code** | **C1** | **C2** | **C3** | **C4** | **C5** |
| --- | --- | --- | --- | --- | --- |
| **D1** | (0.5, 0.6, 0.7, 0.8) | (0.7, 0.8, 0.8, 0.9) | (0.1, 0.2, 0.2, 0.3) | (0.7, 0.8, 0.8, 0.9) | (0.4, 0.5, 0.5, 0.6) |
| **D2** | (0.7, 0.8, 0.8, 0.9) | (0.7, 0.8, 0.8, 0.9) | (0.7, 0.8, 0.8, 0.9) | (0.7, 0.8, 0.8, 0.9) | (0.5, 0.6, 0.7, 0.8) |
| **D3** | (0.7, 0.8, 0.8, 0.9) | (0.8, 0.9, 1, 1) | (0.7, 0.8, 0.8, 0.9) | (0.7, 0.8, 0.8, 0.9) | (0.2, 0.3, 0.4, 0.5) |
| **D4** | (0.8, 0.9, 1, 1) | (0.7, 0.8, 0.8, 0.9) | (0.7, 0.8, 0.8, 0.9) | (0.8, 0.9, 1, 1) | (0.5, 0.6, 0.7, 0.8) |
| **D5** | (0.2, 0.3, 0.4, 0.5) | (0.7, 0.8, 0.8, 0.9) | (0.7, 0.8, 0.8, 0.9) | (0.4, 0.5, 0.5, 0.6) | (0.5, 0.6, 0.7, 0.8) |
| **D6** | (0.8, 0.9, 1, 1) | (0.7, 0.8, 0.8, 0.9) | (0.7, 0.8, 0.8, 0.9) | (0.4, 0.5, 0.5, 0.6) | (0.7, 0.8, 0.8, 0.9) |
| **D7** | (0.5, 0.6, 0.7, 0.8) | (0.7, 0.8, 0.8, 0.9) | (0.7, 0.8, 0.8, 0.9) | (0.5, 0.6, 0.7, 0.8) | (0.4, 0.5, 0.5, 0.6) |
| **D8** | (0.2, 0.3, 0.4, 0.5) | (0.7, 0.8, 0.8, 0.9) | (0.8, 0.9, 1, 1) | (0.4, 0.5, 0.5, 0.6) | (0.7, 0.8, 0.8, 0.9) |
| **D9** | (0.8, 0.9, 1, 1) | (0.7, 0.8, 0.8, 0.9) | (0.7, 0.8, 0.8, 0.9) | (0.4, 0.5, 0.5, 0.6) | (0.8, 0.9, 1, 1) |
| **D10** | (0.7, 0.8, 0.8, 0.9) | (0.8, 0.9, 1, 1) | (0.5, 0.6, 0.7, 0.8) | (0.7, 0.8, 0.8, 0.9) | (0.7, 0.8, 0.8, 0.9) |
| **D11** | (0.8, 0.9, 1, 1) | (0.5, 0.6, 0.7, 0.8) | (0.5, 0.6, 0.7, 0.8) | (0.4, 0.5, 0.5, 0.6) | (0.5, 0.6, 0.7, 0.8) |
| **D12** | (0.1, 0.2, 0.2, 0.3) | (0.4, 0.5, 0.5, 0.6) | (0.7, 0.8, 0.8, 0.9) | (0.2, 0.3, 0.4, 0.5) | (0.5, 0.6, 0.7, 0.8) |
| **D13** | (0.1, 0.2, 0.2, 0.3) | (0.5, 0.6, 0.7, 0.8) | (0.7, 0.8, 0.8, 0.9) | (0.7, 0.8, 0.8, 0.9) | (0.7, 0.8, 0.8, 0.9) |
| **D14** | (0.5, 0.6, 0.7, 0.8) | (0.7, 0.8, 0.8, 0.9) | (0.8, 0.9, 1, 1) | (0.5, 0.6, 0.7, 0.8) | (0.7, 0.8, 0.8, 0.9) |
| **D15** | (0.2, 0.3, 0.4, 0.5) | (0.5, 0.6, 0.7, 0.8) | (0.7, 0.8, 0.8, 0.9) | (0.4, 0.5, 0.5, 0.6) | (0.5, 0.6, 0.7, 0.8) |
| **D16** | (0.5, 0.6, 0.7, 0.8) | (0.5, 0.6, 0.7, 0.8) | (0.7, 0.8, 0.8, 0.9) | (0.7, 0.8, 0.8, 0.9) | (0.1, 0.2, 0.2, 0.3) |
| **D17** | (0.7, 0.8, 0.8, 0.9) | (0.8, 0.9, 1, 1) | (0.7, 0.8, 0.8, 0.9) | (0.5, 0.6, 0.7, 0.8) | (0.2, 0.3, 0.4, 0.5) |
| **D18** | (0.5, 0.6, 0.7, 0.8) | (0.2, 0.3, 0.4, 0.5) | (0.5, 0.6, 0.7, 0.8) | (0.2, 0.3, 0.4, 0.5) | (0.4, 0.5, 0.5, 0.6) |
| **D19** | (0.4, 0.5, 0.5, 0.6) | (0.7, 0.8, 0.8, 0.9) | (0.7, 0.8, 0.8, 0.9) | (0.4, 0.5, 0.5, 0.6) | (0.4, 0.5, 0.5, 0.6) |
| **D20** | (0.7, 0.8, 0.8, 0.9) | (0.4, 0.5, 0.5, 0.6) | (0.8, 0.9, 1, 1) | (0.7, 0.8, 0.8, 0.9) | (0.1, 0.2, 0.2, 0.3) |
| **D21** | (0.8, 0.9, 1, 1) | (0.8, 0.9, 1, 1) | (0.8, 0.9, 1, 1) | (0.7, 0.8, 0.8, 0.9) | (0.1, 0.2, 0.2, 0.3) |
| **D22** | (0.7, 0.8, 0.8, 0.9) | (0.8, 0.9, 1, 1) | (0.8, 0.9, 1, 1) | (0.7, 0.8, 0.8, 0.9) | (0.7, 0.8, 0.8, 0.9) |
| **D23** | (0.5, 0.6, 0.7, 0.8) | (0.7, 0.8, 0.8, 0.9) | (0.8, 0.9, 1, 1) | (0.4, 0.5, 0.5, 0.6) | (0.7, 0.8, 0.8, 0.9) |
| **D24** | (0.1, 0.2, 0.2, 0.3) | (0.2, 0.3, 0.4, 0.5) | (0.1, 0.2, 0.2, 0.3) | (0.4, 0.5, 0.5, 0.6) | (0.8, 0.9, 1, 1) |
| **D25** | (0.8, 0.9, 1, 1) | (0.7, 0.8, 0.8, 0.9) | (0.8, 0.9, 1, 1) | (0.8, 0.9, 1, 1) | (0.4, 0.5, 0.5, 0.6) |
|  | **C6** | **C7** | **C8** | **C9** | **C10** |
| **D1** | (0.5, 0.6, 0.7, 0.8) | (0.5, 0.6, 0.7, 0.8) | (0.8, 0.9, 1, 1) | (0.7, 0.8, 0.8, 0.9) | (0.4, 0.5, 0.5, 0.6) |
| **D2** | (0.5, 0.6, 0.7, 0.8) | (0.5, 0.6, 0.7, 0.8) | (0.8, 0.9, 1, 1) | (0.8, 0.9, 1, 1) | (0.5, 0.6, 0.7, 0.8) |
| **D3** | (0.5, 0.6, 0.7, 0.8) | (0.5, 0.6, 0.7, 0.8) | (0.7, 0.8, 0.8, 0.9) | (0.5, 0.6, 0.7, 0.8) | (0.5, 0.6, 0.7, 0.8) |
| **D4** | (0.7, 0.8, 0.8, 0.9) | (0.2, 0.3, 0.4, 0.5) | (0.5, 0.6, 0.7, 0.8) | (0.4, 0.5, 0.5, 0.6) | (0.2, 0.3, 0.4, 0.5) |
| **D5** | (0.5, 0.6, 0.7, 0.8) | (0.2, 0.3, 0.4, 0.5) | (0.5, 0.6, 0.7, 0.8) | (0.4, 0.5, 0.5, 0.6) | (0.5, 0.6, 0.7, 0.8) |
| **D6** | (0.5, 0.6, 0.7, 0.8) | (0.5, 0.6, 0.7, 0.8) | (0.8, 0.9, 1, 1) | (0.8, 0.9, 1, 1) | (0.5, 0.6, 0.7, 0.8) |
| **D7** | (0.5, 0.6, 0.7, 0.8) | (0.5, 0.6, 0.7, 0.8) | (0.8, 0.9, 1, 1) | (0.4, 0.5, 0.5, 0.6) | (0.5, 0.6, 0.7, 0.8) |
| **D8** | (0.4, 0.5, 0.5, 0.6) | (0.2, 0.3, 0.4, 0.5) | (0.8, 0.9, 1, 1) | (0.7, 0.8, 0.8, 0.9) | (0.1, 0.2, 0.2, 0.3) |
| **D9** | (0.5, 0.6, 0.7, 0.8) | (0.7, 0.8, 0.8, 0.9) | (0.8, 0.9, 1, 1) | (0.5, 0.6, 0.7, 0.8) | (0.4, 0.5, 0.5, 0.6) |
| **D10** | (0.4, 0.5, 0.5, 0.6) | (0.2, 0.3, 0.4, 0.5) | (0.7, 0.8, 0.8, 0.9) | (0.4, 0.5, 0.5, 0.6) | (0.4, 0.5, 0.5, 0.6) |
| **D11** | (0.5, 0.6, 0.7, 0.8) | (0.2, 0.3, 0.4, 0.5) | (0.8, 0.9, 1, 1) | (0.5, 0.6, 0.7, 0.8) | (0.4, 0.5, 0.5, 0.6) |
| **D12** | (0.4, 0.5, 0.5, 0.6) | (0.2, 0.3, 0.4, 0.5) | (0.8, 0.9, 1, 1) | (0.5, 0.6, 0.7, 0.8) | (0.5, 0.6, 0.7, 0.8) |
| **D13** | (0.4, 0.5, 0.5, 0.6) | (0.4, 0.5, 0.5, 0.6) | (0.8, 0.9, 1, 1) | (0.7, 0.8, 0.8, 0.9) | (0.7, 0.8, 0.8, 0.9) |
| **D14** | (0.5, 0.6, 0.7, 0.8) | (0.2, 0.3, 0.4, 0.5) | (0.5, 0.6, 0.7, 0.8) | (0.5, 0.6, 0.7, 0.8) | (0.4, 0.5, 0.5, 0.6) |
| **D15** | (0.4, 0.5, 0.5, 0.6) | (0.4, 0.5, 0.5, 0.6) | (0.7, 0.8, 0.8, 0.9) | (0.5, 0.6, 0.7, 0.8) | (0.2, 0.3, 0.4, 0.5) |
| **D16** | (0.7, 0.8, 0.8, 0.9) | (0.4, 0.5, 0.5, 0.6) | (0.8, 0.9, 1, 1) | (0.5, 0.6, 0.7, 0.8) | (0.2, 0.3, 0.4, 0.5) |
| **D17** | (0.4, 0.5, 0.5, 0.6) | (0.5, 0.6, 0.7, 0.8) | (0.8, 0.9, 1, 1) | (0.7, 0.8, 0.8, 0.9) | (0.2, 0.3, 0.4, 0.5) |
| **D18** | (0.5, 0.6, 0.7, 0.8) | (0.4, 0.5, 0.5, 0.6) | (0.8, 0.9, 1, 1) | (0.5, 0.6, 0.7, 0.8) | (0.5, 0.6, 0.7, 0.8) |
| **D19** | (0.5, 0.6, 0.7, 0.8) | (0.2, 0.3, 0.4, 0.5) | (0.8, 0.9, 1, 1) | (0.5, 0.6, 0.7, 0.8) | (0, 0, 0.1, 0.2) |
| **D20** | (0.7, 0.8, 0.8, 0.9) | (0.1, 0.2, 0.2, 0.3) | (0.8, 0.9, 1, 1) | (0.8, 0.9, 1, 1) | (0.2, 0.3, 0.4, 0.5) |
| **D21** | (0.5, 0.6, 0.7, 0.8) | (0.7, 0.8, 0.8, 0.9) | (0.7, 0.8, 0.8, 0.9) | (0.8, 0.9, 1, 1) | (0.7, 0.8, 0.8, 0.9) |
| **D22** | (0.4, 0.5, 0.5, 0.6) | (0.5, 0.6, 0.7, 0.8) | (0.7, 0.8, 0.8, 0.9) | (0.8, 0.9, 1, 1) | (0.7, 0.8, 0.8, 0.9) |
| **D23** | (0.5, 0.6, 0.7, 0.8) | (0.7, 0.8, 0.8, 0.9) | (0.7, 0.8, 0.8, 0.9) | (0.5, 0.6, 0.7, 0.8) | (0.2, 0.3, 0.4, 0.5) |
| **D24** | (0.5, 0.6, 0.7, 0.8) | (0.1, 0.2, 0.2, 0.3) | (0.4, 0.5, 0.5, 0.6) | (0.4, 0.5, 0.5, 0.6) | (0.2, 0.3, 0.4, 0.5) |
| **D25** | (0.5, 0.6, 0.7, 0.8) | (0.4, 0.5, 0.5, 0.6) | (0.7, 0.8, 0.8, 0.9) | (0.5, 0.6, 0.7, 0.8) | (0.2, 0.3, 0.4, 0.5) |

**Table G. Objective weights and subjective weights**

| **Code** | **Objective weights** | **Subjective weights** | **Subjective weights (Defuzzified)** |
| --- | --- | --- | --- |
| **D1** | 0.048366 | (0.44, 0.54, 0.58, 0.67) | 0.556914 |
| **D2** | 0.020132 | (0.73, 0.83, 0.89, 0.94) | 0.845185 |
| **D3** | 0.037226 | (0.62, 0.72, 0.79, 0.85) | 0.743222 |
| **D4** | 0.044638 | (0.58, 0.68, 0.74, 0.82) | 0.704000 |
| **D5** | 0.033306 | (0.56, 0.66, 0.73, 0.81) | 0.689063 |
| **D6** | 0.031088 | (0.75, 0.85, 0.9, 0.95) | 0.860000 |
| **D7** | 0.030651 | (0.65, 0.75, 0.82, 0.87) | 0.770345 |
| **D8** | 0.03587 | (0.7, 0.8, 0.86, 0.91) | 0.815185 |
| **D9** | 0.029671 | (0.75, 0.85, 0.93, 0.96) | 0.869885 |
| **D10** | 0.034036 | (0.66, 0.76, 0.81, 0.88) | 0.775926 |
| **D11** | 0.046591 | (0.67, 0.77, 0.8, 0.88) | 0.778750 |
| **D12** | 0.042575 | (0.49, 0.59, 0.65, 0.74) | 0.616989 |
| **D13** | 0.046805 | (0.6, 0.7, 0.75, 0.83) | 0.718929 |
| **D14** | 0.041398 | (0.67, 0.77, 0.83, 0.89) | 0.788095 |
| **D15** | 0.027059 | (0.62, 0.72, 0.79, 0.85) | 0.743222 |
| **D16** | 0.044898 | (0.59, 0.69, 0.73, 0.79) | 0.697778 |
| **D17** | 0.03942 | (0.64, 0.74, 0.8, 0.87) | 0.761034 |
| **D18** | 0.040199 | (0.37, 0.47, 0.53, 0.62) | 0.496989 |
| **D19** | 0.049852 | (0.63, 0.73, 0.78, 0.85) | 0.745926 |
| **D20** | 0.043022 | (0.53, 0.63, 0.67, 0.73) | 0.637778 |
| **D21** | 0.06917 | (0.64, 0.74, 0.8, 0.84) | 0.752308 |
| **D22** | 0.045785 | (0.67, 0.77, 0.83, 0.88) | 0.785185 |
| **D23** | 0.034187 | (0.53, 0.63, 0.67, 0.76) | 0.646914 |
| **D24** | 0.050747 | (0.43, 0.53, 0.56, 0.65) | 0.541867 |
| **D25** | 0.033308 | (0.57, 0.67, 0.72, 0.79) | 0.685926 |

**Table H.** **Aggregated weights.**

| **Code** | **Aggregated weights** | | **Rank** |
| --- | --- | --- | --- |
|  | **Fuzzy** | **De-fuzzy** |  |
| **D1** | (0.24, 0.29, 0.31, 0.36) | 0.30264 | 23 |
| **D2** | (0.38, 0.43, 0.46, 0.48) | 0.432658 | 3 |
| **D3** | (0.33, 0.38, 0.41, 0.44) | 0.390224 | 13 |
| **D4** | (0.31, 0.36, 0.39, 0.43) | 0.374319 | 16 |
| **D5** | (0.3, 0.35, 0.38, 0.42) | 0.361184 | 18 |
| **D6** | (0.39, 0.44, 0.47, 0.49) | 0.445544 | 2 |
| **D7** | (0.34, 0.39, 0.43, 0.45) | 0.400498 | 10 |
| **D8** | (0.37, 0.42, 0.45, 0.47) | 0.425527 | 4 |
| **D9** | (0.39, 0.44, 0.48, 0.49) | 0.449778 | 1 |
| **D10** | (0.35, 0.4, 0.42, 0.46) | 0.404981 | 9 |
| **D11** | (0.36, 0.41, 0.42, 0.46) | 0.412671 | 7 |
| **D12** | (0.27, 0.32, 0.35, 0.39) | 0.329782 | 22 |
| **D13** | (0.32, 0.37, 0.4, 0.44) | 0.382867 | 15 |
| **D14** | (0.36, 0.41, 0.44, 0.47) | 0.414747 | 6 |
| **D15** | (0.32, 0.37, 0.41, 0.44) | 0.385141 | 14 |
| **D16** | (0.32, 0.37, 0.39, 0.42) | 0.371338 | 20 |
| **D17** | (0.34, 0.39, 0.42, 0.45) | 0.400227 | 17 |
| **D18** | (0.21, 0.26, 0.29, 0.33) | 0.268594 | 25 |
| **D19** | (0.34, 0.39, 0.41, 0.45) | 0.397889 | 12 |
| **D20** | (0.29, 0.34, 0.36, 0.39) | 0.3404 | 21 |
| **D21** | (0.35, 0.4, 0.43, 0.45) | 0.410739 | 8 |
| **D22** | (0.36, 0.41, 0.44, 0.46) | 0.415485 | 5 |
| **D23** | (0.28, 0.33, 0.35, 0.4) | 0.34055 | 20 |
| **D24** | (0.24, 0.29, 0.31, 0.35) | 0.296307 | 24 |
| **D25** | (0.3, 0.35, 0.38, 0.41) | 0.359617 | 19 |

**Table I.** **Standard deviation and rank.**

| **Determinant Code** | **Standard Deviation** | **Rank** |
| --- | --- | --- |
| **D1** | 0.199186 | 11 |
| **D2** | 0.107703 | 25 |
| **D3** | 0.15141 | 21 |
| **D4** | 0.205426 | 9 |
| **D5** | 0.154919 | 19 |
| **D6** | 0.140446 | 22 |
| **D7** | 0.128355 | 23 |
| **D8** | 0.252982 | 2 |
| **D9** | 0.158351 | 17 |
| **D10** | 0.180762 | 13 |
| **D11** | 0.17088 | 14 |
| **D12** | 0.211365 | 6 |
| **D13** | 0.207786 | 8 |
| **D14** | 0.154191 | 20 |
| **D15** | 0.155724 | 18 |
| **D16** | 0.214884 | 5 |
| **D17** | 0.205426 | 10 |
| **D18** | 0.163325 | 15 |
| **D19** | 0.236538 | 3 |
| **D20** | 0.285788 | 1 |
| **D21** | 0.210832 | 7 |
| **D22** | 0.12816 | 24 |
| **D23** | 0.163325 | 16 |
| **D24** | 0.220284 | 4 |
| **D25** | 0.196532 | 12 |

**Table J.** **Matrix of the normalized decision.**

| **Code** | **C1** | **C2** | **C3** | **C4** | **C5** |
| --- | --- | --- | --- | --- | --- |
| **D1** | (0.5, 0.6, 0.7, 0.8) | (0.7, 0.8, 0.8, 0.9) | (0.1, 0.2, 0.2, 0.3) | (0.7, 0.8, 0.8, 0.9) | (0.4, 0.5, 0.5, 0.6) |
| **D2** | (0.7, 0.8, 0.8, 0.9) | (0.7, 0.8, 0.8, 0.9) | (0.7, 0.8, 0.8, 0.9) | (0.7, 0.8, 0.8, 0.9) | (0.5, 0.6, 0.7, 0.8) |
| **D3** | (0.7, 0.8, 0.8, 0.9) | (0.8, 0.9, 1, 1) | (0.7, 0.8, 0.8, 0.9) | (0.7, 0.8, 0.8, 0.9) | (0.2, 0.3, 0.4, 0.5) |
| **D4** | (0.8, 0.9, 1, 1) | (0.7, 0.8, 0.8, 0.9) | (0.7, 0.8, 0.8, 0.9) | (0.8, 0.9, 1, 1) | (0.5, 0.6, 0.7, 0.8) |
| **D5** | (0.22, 0.33, 0.44, 0.56) | (0.78, 0.89, 0.89, 1) | (0.78, 0.89, 0.89, 1) | (0.44, 0.56, 0.56, 0.67) | (0.56, 0.67, 0.78, 0.89) |
| **D6** | (0.8, 0.9, 1, 1) | (0.7, 0.8, 0.8, 0.9) | (0.7, 0.8, 0.8, 0.9) | (0.4, 0.5, 0.5, 0.6) | (0.7, 0.8, 0.8, 0.9) |
| **D7** | (0.5, 0.6, 0.7, 0.8) | (0.7, 0.8, 0.8, 0.9) | (0.7, 0.8, 0.8, 0.9) | (0.5, 0.6, 0.7, 0.8) | (0.4, 0.5, 0.5, 0.6) |
| **D8** | (0.2, 0.3, 0.4, 0.5) | (0.7, 0.8, 0.8, 0.9) | (0.8, 0.9, 1, 1) | (0.4, 0.5, 0.5, 0.6) | (0.7, 0.8, 0.8, 0.9) |
| **D9** | (0.8, 0.9, 1, 1) | (0.7, 0.8, 0.8, 0.9) | (0.7, 0.8, 0.8, 0.9) | (0.4, 0.5, 0.5, 0.6) | (0.8, 0.9, 1, 1) |
| **D10** | (0.7, 0.8, 0.8, 0.9) | (0.8, 0.9, 1, 1) | (0.5, 0.6, 0.7, 0.8) | (0.7, 0.8, 0.8, 0.9) | (0.7, 0.8, 0.8, 0.9) |
| **D11** | (0.8, 0.9, 1, 1) | (0.5, 0.6, 0.7, 0.8) | (0.5, 0.6, 0.7, 0.8) | (0.4, 0.5, 0.5, 0.6) | (0.5, 0.6, 0.7, 0.8) |
| **D12** | (0.1, 0.2, 0.2, 0.3) | (0.4, 0.5, 0.5, 0.6) | (0.7, 0.8, 0.8, 0.9) | (0.2, 0.3, 0.4, 0.5) | (0.5, 0.6, 0.7, 0.8) |
| **D13** | (0.1, 0.2, 0.2, 0.3) | (0.5, 0.6, 0.7, 0.8) | (0.7, 0.8, 0.8, 0.9) | (0.7, 0.8, 0.8, 0.9) | (0.7, 0.8, 0.8, 0.9) |
| **D14** | (0.5, 0.6, 0.7, 0.8) | (0.7, 0.8, 0.8, 0.9) | (0.8, 0.9, 1, 1) | (0.5, 0.6, 0.7, 0.8) | (0.7, 0.8, 0.8, 0.9) |
| **D15** | (0.22, 0.33, 0.44, 0.56) | (0.56, 0.67, 0.78, 0.89) | (0.78, 0.89, 0.89, 1) | (0.44, 0.56, 0.56, 0.67) | (0.56, 0.67, 0.78, 0.89) |
| **D16** | (0.13, 0.14, 0.17, 0.2) | (0.13, 0.14, 0.17, 0.2) | (0.11, 0.13, 0.13, 0.14) | (0.11, 0.13, 0.13, 0.14) | (0.33, 0.5, 0.5, 1) |
| **D17** | (0.22, 0.25, 0.25, 0.29) | (0.2, 0.2, 0.22, 0.25) | (0.22, 0.25, 0.25, 0.29) | (0.25, 0.29, 0.33, 0.4) | (0.4, 0.5, 0.67, 1) |
| **D18** | (0.5, 0.6, 0.7, 0.8) | (0.2, 0.3, 0.4, 0.5) | (0.5, 0.6, 0.7, 0.8) | (0.2, 0.3, 0.4, 0.5) | (0.4, 0.5, 0.5, 0.6) |
| **D19** | (0.4, 0.5, 0.5, 0.6) | (0.7, 0.8, 0.8, 0.9) | (0.7, 0.8, 0.8, 0.9) | (0.4, 0.5, 0.5, 0.6) | (0.4, 0.5, 0.5, 0.6) |
| **D20** | (0.7, 0.8, 0.8, 0.9) | (0.4, 0.5, 0.5, 0.6) | (0.8, 0.9, 1, 1) | (0.7, 0.8, 0.8, 0.9) | (0.1, 0.2, 0.2, 0.3) |
| **D21** | (0.8, 0.9, 1, 1) | (0.8, 0.9, 1, 1) | (0.8, 0.9, 1, 1) | (0.7, 0.8, 0.8, 0.9) | (0.1, 0.2, 0.2, 0.3) |
| **D22** | (0.7, 0.8, 0.8, 0.9) | (0.8, 0.9, 1, 1) | (0.8, 0.9, 1, 1) | (0.7, 0.8, 0.8, 0.9) | (0.7, 0.8, 0.8, 0.9) |
| **D23** | (0.25, 0.29, 0.33, 0.4) | (0.22, 0.25, 0.25, 0.29) | (0.2, 0.2, 0.22, 0.25) | (0.33, 0.4, 0.4, 0.5) | (0.22, 0.25, 0.25, 0.29) |
| **D24** | (0.1, 0.2, 0.2, 0.3) | (0.2, 0.3, 0.4, 0.5) | (0.1, 0.2, 0.2, 0.3) | (0.4, 0.5, 0.5, 0.6) | (0.8, 0.9, 1, 1) |
| **D25** | (0.8, 0.9, 1, 1) | (0.7, 0.8, 0.8, 0.9) | (0.8, 0.9, 1, 1) | (0.8, 0.9, 1, 1) | (0.4, 0.5, 0.5, 0.6) |
|  | **C6** | **C7** | **C8** | **C9** | **C10** |
| **D1** | (0.5, 0.6, 0.7, 0.8) | (0.5, 0.6, 0.7, 0.8) | (0.8, 0.9, 1, 1) | (0.7, 0.8, 0.8, 0.9) | (0.4, 0.5, 0.5, 0.6) |
| **D2** | (0.5, 0.6, 0.7, 0.8) | (0.5, 0.6, 0.7, 0.8) | (0.8, 0.9, 1, 1) | (0.8, 0.9, 1, 1) | (0.5, 0.6, 0.7, 0.8) |
| **D3** | (0.5, 0.6, 0.7, 0.8) | (0.5, 0.6, 0.7, 0.8) | (0.7, 0.8, 0.8, 0.9) | (0.5, 0.6, 0.7, 0.8) | (0.5, 0.6, 0.7, 0.8) |
| **D4** | (0.7, 0.8, 0.8, 0.9) | (0.2, 0.3, 0.4, 0.5) | (0.5, 0.6, 0.7, 0.8) | (0.4, 0.5, 0.5, 0.6) | (0.2, 0.3, 0.4, 0.5) |
| **D5** | (0.56, 0.67, 0.78, 0.89) | (0.22, 0.33, 0.44, 0.56) | (0.56, 0.67, 0.78, 0.89) | (0.44, 0.56, 0.56, 0.67) | (0.56, 0.67, 0.78, 0.89) |
| **D6** | (0.5, 0.6, 0.7, 0.8) | (0.5, 0.6, 0.7, 0.8) | (0.8, 0.9, 1, 1) | (0.8, 0.9, 1, 1) | (0.5, 0.6, 0.7, 0.8) |
| **D7** | (0.5, 0.6, 0.7, 0.8) | (0.5, 0.6, 0.7, 0.8) | (0.8, 0.9, 1, 1) | (0.4, 0.5, 0.5, 0.6) | (0.5, 0.6, 0.7, 0.8) |
| **D8** | (0.4, 0.5, 0.5, 0.6) | (0.2, 0.3, 0.4, 0.5) | (0.8, 0.9, 1, 1) | (0.7, 0.8, 0.8, 0.9) | (0.1, 0.2, 0.2, 0.3) |
| **D9** | (0.5, 0.6, 0.7, 0.8) | (0.7, 0.8, 0.8, 0.9) | (0.8, 0.9, 1, 1) | (0.5, 0.6, 0.7, 0.8) | (0.4, 0.5, 0.5, 0.6) |
| **D10** | (0.4, 0.5, 0.5, 0.6) | (0.2, 0.3, 0.4, 0.5) | (0.7, 0.8, 0.8, 0.9) | (0.4, 0.5, 0.5, 0.6) | (0.4, 0.5, 0.5, 0.6) |
| **D11** | (0.5, 0.6, 0.7, 0.8) | (0.2, 0.3, 0.4, 0.5) | (0.8, 0.9, 1, 1) | (0.5, 0.6, 0.7, 0.8) | (0.4, 0.5, 0.5, 0.6) |
| **D12** | (0.4, 0.5, 0.5, 0.6) | (0.2, 0.3, 0.4, 0.5) | (0.8, 0.9, 1, 1) | (0.5, 0.6, 0.7, 0.8) | (0.5, 0.6, 0.7, 0.8) |
| **D13** | (0.4, 0.5, 0.5, 0.6) | (0.4, 0.5, 0.5, 0.6) | (0.8, 0.9, 1, 1) | (0.7, 0.8, 0.8, 0.9) | (0.7, 0.8, 0.8, 0.9) |
| **D14** | (0.5, 0.6, 0.7, 0.8) | (0.2, 0.3, 0.4, 0.5) | (0.5, 0.6, 0.7, 0.8) | (0.5, 0.6, 0.7, 0.8) | (0.4, 0.5, 0.5, 0.6) |
| **D15** | (0.44, 0.56, 0.56, 0.67) | (0.44, 0.56, 0.56, 0.67) | (0.78, 0.89, 0.89, 1) | (0.56, 0.67, 0.78, 0.89) | (0.22, 0.33, 0.44, 0.56) |
| **D16** | (0.11, 0.13, 0.13, 0.14) | (0.17, 0.2, 0.2, 0.25) | (0.1, 0.1, 0.11, 0.13) | (0.13, 0.14, 0.17, 0.2) | (0.2, 0.25, 0.33, 0.5) |
| **D17** | (0.33, 0.4, 0.4, 0.5) | (0.25, 0.29, 0.33, 0.4) | (0.2, 0.2, 0.22, 0.25) | (0.22, 0.25, 0.25, 0.29) | (0.4, 0.5, 0.67, 1) |
| **D18** | (0.5, 0.6, 0.7, 0.8) | (0.4, 0.5, 0.5, 0.6) | (0.8, 0.9, 1, 1) | (0.5, 0.6, 0.7, 0.8) | (0.5, 0.6, 0.7, 0.8) |
| **D19** | (0.5, 0.6, 0.7, 0.8) | (0.2, 0.3, 0.4, 0.5) | (0.8, 0.9, 1, 1) | (0.5, 0.6, 0.7, 0.8) | (0, 0, 0.1, 0.2) |
| **D20** | (0.7, 0.8, 0.8, 0.9) | (0.1, 0.2, 0.2, 0.3) | (0.8, 0.9, 1, 1) | (0.8, 0.9, 1, 1) | (0.2, 0.3, 0.4, 0.5) |
| **D21** | (0.5, 0.6, 0.7, 0.8) | (0.7, 0.8, 0.8, 0.9) | (0.7, 0.8, 0.8, 0.9) | (0.8, 0.9, 1, 1) | (0.7, 0.8, 0.8, 0.9) |
| **D22** | (0.4, 0.5, 0.5, 0.6) | (0.5, 0.6, 0.7, 0.8) | (0.7, 0.8, 0.8, 0.9) | (0.8, 0.9, 1, 1) | (0.7, 0.8, 0.8, 0.9) |
| **D23** | (0.25, 0.29, 0.33, 0.4) | (0.22, 0.25, 0.25, 0.29) | (0.22, 0.25, 0.25, 0.29) | (0.25, 0.29, 0.33, 0.4) | (0.4, 0.5, 0.67, 1) |
| **D24** | (0.5, 0.6, 0.7, 0.8) | (0.1, 0.2, 0.2, 0.3) | (0.4, 0.5, 0.5, 0.6) | (0.4, 0.5, 0.5, 0.6) | (0.2, 0.3, 0.4, 0.5) |
| **D25** | (0.5, 0.6, 0.7, 0.8) | (0.4, 0.5, 0.5, 0.6) | (0.7, 0.8, 0.8, 0.9) | (0.5, 0.6, 0.7, 0.8) | (0.2, 0.3, 0.4, 0.5) |

**Table K.** **Matrix of the weighted normalized decision.**

| **Code** | **C1** | **C2** | **C3** | **C4** | **C5** |
| --- | --- | --- | --- | --- | --- |
| **D1** | (0.12, 0.18, 0.22, 0.29) | (0.17, 0.24, 0.25, 0.32) | (0.02, 0.06, 0.06, 0.11) | (0.17, 0.24, 0.25, 0.32) | (0.1, 0.15, 0.16, 0.22) |
| **D2** | (0.26, 0.34, 0.36, 0.43) | (0.26, 0.34, 0.36, 0.43) | (0.26, 0.34, 0.36, 0.43) | (0.26, 0.34, 0.36, 0.43) | (0.19, 0.26, 0.32, 0.38) |
| **D3** | (0.23, 0.3, 0.33, 0.4) | (0.26, 0.34, 0.41, 0.44) | (0.23, 0.3, 0.33, 0.4) | (0.23, 0.3, 0.33, 0.4) | (0.07, 0.11, 0.17, 0.22) |
| **D4** | (0.25, 0.33, 0.39, 0.43) | (0.22, 0.29, 0.31, 0.39) | (0.22, 0.29, 0.31, 0.39) | (0.25, 0.33, 0.39, 0.43) | (0.16, 0.22, 0.27, 0.35) |
| **D5** | (0.07, 0.11, 0.17, 0.24) | (0.23, 0.31, 0.34, 0.42) | (0.23, 0.31, 0.34, 0.42) | (0.13, 0.19, 0.21, 0.28) | (0.17, 0.23, 0.3, 0.38) |
| **D6** | (0.31, 0.4, 0.47, 0.49) | (0.27, 0.35, 0.37, 0.44) | (0.27, 0.35, 0.37, 0.44) | (0.16, 0.22, 0.23, 0.29) | (0.27, 0.35, 0.37, 0.44) |
| **D7** | (0.17, 0.23, 0.3, 0.36) | (0.24, 0.31, 0.34, 0.41) | (0.24, 0.31, 0.34, 0.41) | (0.17, 0.23, 0.3, 0.36) | (0.14, 0.2, 0.21, 0.27) |
| **D8** | (0.07, 0.13, 0.18, 0.24) | (0.26, 0.33, 0.36, 0.43) | (0.29, 0.38, 0.45, 0.47) | (0.15, 0.21, 0.22, 0.28) | (0.26, 0.33, 0.36, 0.43) |
| **D9** | (0.31, 0.4, 0.48, 0.49) | (0.27, 0.35, 0.38, 0.45) | (0.27, 0.35, 0.38, 0.45) | (0.16, 0.22, 0.24, 0.3) | (0.31, 0.4, 0.48, 0.49) |
| **D10** | (0.24, 0.32, 0.34, 0.41) | (0.28, 0.36, 0.42, 0.46) | (0.17, 0.24, 0.3, 0.37) | (0.24, 0.32, 0.34, 0.41) | (0.24, 0.32, 0.34, 0.41) |
| **D11** | (0.29, 0.37, 0.42, 0.46) | (0.18, 0.24, 0.3, 0.37) | (0.18, 0.24, 0.3, 0.37) | (0.14, 0.2, 0.21, 0.28) | (0.18, 0.24, 0.3, 0.37) |
| **D12** | (0.03, 0.06, 0.07, 0.12) | (0.11, 0.16, 0.17, 0.23) | (0.19, 0.25, 0.28, 0.35) | (0.05, 0.09, 0.14, 0.2) | (0.13, 0.19, 0.24, 0.31) |
| **D13** | (0.03, 0.07, 0.08, 0.13) | (0.16, 0.22, 0.28, 0.35) | (0.23, 0.3, 0.32, 0.39) | (0.23, 0.3, 0.32, 0.39) | (0.23, 0.3, 0.32, 0.39) |
| **D14** | (0.18, 0.24, 0.3, 0.37) | (0.25, 0.32, 0.35, 0.42) | (0.28, 0.37, 0.44, 0.47) | (0.18, 0.24, 0.3, 0.37) | (0.25, 0.32, 0.35, 0.42) |
| **D15** | (0.07, 0.12, 0.18, 0.25) | (0.18, 0.25, 0.32, 0.39) | (0.25, 0.33, 0.36, 0.44) | (0.14, 0.21, 0.23, 0.29) | (0.18, 0.25, 0.32, 0.39) |
| **D16** | (0.04, 0.05, 0.07, 0.08) | (0.04, 0.05, 0.07, 0.08) | (0.03, 0.05, 0.05, 0.06) | (0.03, 0.05, 0.05, 0.06) | (0.1, 0.18, 0.19, 0.42) |
| **D17** | (0.07, 0.1, 0.1, 0.13) | (0.07, 0.08, 0.09, 0.11) | (0.07, 0.1, 0.1, 0.13) | (0.08, 0.11, 0.14, 0.18) | (0.14, 0.19, 0.28, 0.45) |
| **D18** | (0.1, 0.15, 0.2, 0.26) | (0.04, 0.08, 0.11, 0.17) | (0.1, 0.15, 0.2, 0.26) | (0.04, 0.08, 0.11, 0.17) | (0.08, 0.13, 0.14, 0.2) |
| **D19** | (0.14, 0.19, 0.21, 0.27) | (0.24, 0.31, 0.33, 0.4) | (0.24, 0.31, 0.33, 0.4) | (0.14, 0.19, 0.21, 0.27) | (0.14, 0.19, 0.21, 0.27) |
| **D20** | (0.2, 0.27, 0.29, 0.35) | (0.11, 0.17, 0.18, 0.23) | (0.23, 0.3, 0.36, 0.39) | (0.2, 0.27, 0.29, 0.35) | (0.03, 0.07, 0.07, 0.12) |
| **D21** | (0.28, 0.36, 0.43, 0.45) | (0.28, 0.36, 0.43, 0.45) | (0.28, 0.36, 0.43, 0.45) | (0.25, 0.32, 0.35, 0.41) | (0.04, 0.08, 0.09, 0.14) |
| **D22** | (0.25, 0.33, 0.35, 0.42) | (0.29, 0.37, 0.44, 0.46) | (0.29, 0.37, 0.44, 0.46) | (0.25, 0.33, 0.35, 0.42) | (0.25, 0.33, 0.35, 0.42) |
| **D23** | (0.07, 0.1, 0.12, 0.16) | (0.06, 0.08, 0.09, 0.12) | (0.06, 0.07, 0.08, 0.1) | (0.09, 0.13, 0.14, 0.2) | (0.06, 0.08, 0.09, 0.12) |
| **D24** | (0.02, 0.06, 0.06, 0.11) | (0.05, 0.09, 0.12, 0.18) | (0.02, 0.06, 0.06, 0.11) | (0.1, 0.15, 0.15, 0.21) | (0.19, 0.26, 0.31, 0.35) |
| **D25** | (0.24, 0.32, 0.38, 0.41) | (0.21, 0.28, 0.3, 0.37) | (0.24, 0.32, 0.38, 0.41) | (0.24, 0.32, 0.38, 0.41) | (0.12, 0.18, 0.19, 0.25) |
|  | **C6** | **C7** | **C8** | **C9** | **C10** |
| **D1** | (0.12, 0.18, 0.22, 0.29) | (0.12, 0.18, 0.22, 0.29) | (0.2, 0.26, 0.31, 0.36) | (0.17, 0.24, 0.25, 0.32) | (0.1, 0.15, 0.16, 0.22) |
| **D2** | (0.19, 0.26, 0.32, 0.38) | (0.19, 0.26, 0.32, 0.38) | (0.3, 0.38, 0.46, 0.48) | (0.3, 0.38, 0.46, 0.48) | (0.19, 0.26, 0.32, 0.38) |
| **D3** | (0.16, 0.23, 0.29, 0.35) | (0.16, 0.23, 0.29, 0.35) | (0.23, 0.3, 0.33, 0.4) | (0.16, 0.23, 0.29, 0.35) | (0.16, 0.23, 0.29, 0.35) |
| **D4** | (0.22, 0.29, 0.31, 0.39) | (0.06, 0.11, 0.16, 0.22) | (0.16, 0.22, 0.27, 0.35) | (0.12, 0.18, 0.2, 0.26) | (0.06, 0.11, 0.16, 0.22) |
| **D5** | (0.17, 0.23, 0.3, 0.38) | (0.07, 0.11, 0.17, 0.24) | (0.17, 0.23, 0.3, 0.38) | (0.13, 0.19, 0.21, 0.28) | (0.17, 0.23, 0.3, 0.38) |
| **D6** | (0.2, 0.26, 0.33, 0.39) | (0.2, 0.26, 0.33, 0.39) | (0.31, 0.4, 0.47, 0.49) | (0.31, 0.4, 0.47, 0.49) | (0.2, 0.26, 0.33, 0.39) |
| **D7** | (0.17, 0.23, 0.3, 0.36) | (0.17, 0.23, 0.3, 0.36) | (0.27, 0.35, 0.43, 0.45) | (0.14, 0.2, 0.21, 0.27) | (0.17, 0.23, 0.3, 0.36) |
| **D8** | (0.15, 0.21, 0.22, 0.28) | (0.07, 0.13, 0.18, 0.24) | (0.29, 0.38, 0.45, 0.47) | (0.26, 0.33, 0.36, 0.43) | (0.04, 0.08, 0.09, 0.14) |
| **D9** | (0.19, 0.26, 0.34, 0.4) | (0.27, 0.35, 0.38, 0.45) | (0.31, 0.4, 0.48, 0.49) | (0.19, 0.26, 0.34, 0.4) | (0.16, 0.22, 0.24, 0.3) |
| **D10** | (0.14, 0.2, 0.21, 0.27) | (0.07, 0.12, 0.17, 0.23) | (0.24, 0.32, 0.34, 0.41) | (0.14, 0.2, 0.21, 0.27) | (0.14, 0.2, 0.21, 0.27) |
| **D11** | (0.18, 0.24, 0.3, 0.37) | (0.07, 0.12, 0.17, 0.23) | (0.29, 0.37, 0.42, 0.46) | (0.18, 0.24, 0.3, 0.37) | (0.14, 0.2, 0.21, 0.28) |
| **D12** | (0.11, 0.16, 0.17, 0.23) | (0.05, 0.09, 0.14, 0.2) | (0.21, 0.28, 0.35, 0.39) | (0.13, 0.19, 0.24, 0.31) | (0.13, 0.19, 0.24, 0.31) |
| **D13** | (0.13, 0.19, 0.2, 0.26) | (0.13, 0.19, 0.2, 0.26) | (0.26, 0.34, 0.4, 0.44) | (0.23, 0.3, 0.32, 0.39) | (0.23, 0.3, 0.32, 0.39) |
| **D14** | (0.18, 0.24, 0.3, 0.37) | (0.07, 0.12, 0.17, 0.23) | (0.18, 0.24, 0.3, 0.37) | (0.18, 0.24, 0.3, 0.37) | (0.14, 0.2, 0.22, 0.28) |
| **D15** | (0.14, 0.21, 0.23, 0.29) | (0.14, 0.21, 0.23, 0.29) | (0.25, 0.33, 0.36, 0.44) | (0.18, 0.25, 0.32, 0.39) | (0.07, 0.12, 0.18, 0.25) |
| **D16** | (0.03, 0.05, 0.05, 0.06) | (0.05, 0.07, 0.08, 0.1) | (0.03, 0.04, 0.04, 0.05) | (0.04, 0.05, 0.07, 0.08) | (0.06, 0.09, 0.13, 0.21) |
| **D17** | (0.11, 0.16, 0.17, 0.23) | (0.08, 0.11, 0.14, 0.18) | (0.07, 0.08, 0.09, 0.11) | (0.07, 0.1, 0.1, 0.13) | (0.14, 0.19, 0.28, 0.45) |
| **D18** | (0.1, 0.15, 0.2, 0.26) | (0.08, 0.13, 0.14, 0.2) | (0.16, 0.23, 0.29, 0.33) | (0.1, 0.15, 0.2, 0.26) | (0.1, 0.15, 0.2, 0.26) |
| **D19** | (0.17, 0.23, 0.29, 0.36) | (0.07, 0.12, 0.17, 0.22) | (0.27, 0.35, 0.41, 0.45) | (0.17, 0.23, 0.29, 0.36) | (0, 0, 0.04, 0.09) |
| **D20** | (0.2, 0.27, 0.29, 0.35) | (0.03, 0.07, 0.07, 0.12) | (0.23, 0.3, 0.36, 0.39) | (0.23, 0.3, 0.36, 0.39) | (0.06, 0.1, 0.14, 0.19) |
| **D21** | (0.18, 0.24, 0.3, 0.36) | (0.25, 0.32, 0.35, 0.41) | (0.25, 0.32, 0.35, 0.41) | (0.28, 0.36, 0.43, 0.45) | (0.25, 0.32, 0.35, 0.41) |
| **D22** | (0.14, 0.2, 0.22, 0.28) | (0.18, 0.24, 0.31, 0.37) | (0.25, 0.33, 0.35, 0.42) | (0.29, 0.37, 0.44, 0.46) | (0.25, 0.33, 0.35, 0.42) |
| **D23** | (0.07, 0.1, 0.12, 0.16) | (0.06, 0.08, 0.09, 0.12) | (0.06, 0.08, 0.09, 0.12) | (0.07, 0.1, 0.12, 0.16) | (0.11, 0.17, 0.24, 0.4) |
| **D24** | (0.12, 0.17, 0.21, 0.28) | (0.02, 0.06, 0.06, 0.11) | (0.1, 0.15, 0.15, 0.21) | (0.1, 0.15, 0.15, 0.21) | (0.05, 0.09, 0.12, 0.18) |
| **D25** | (0.15, 0.21, 0.26, 0.33) | (0.12, 0.18, 0.19, 0.25) | (0.21, 0.28, 0.3, 0.37) | (0.15, 0.21, 0.26, 0.33) | (0.06, 0.11, 0.15, 0.21) |

**Table L. FPIS and distance of each alternative from FPIS.**

| **Code** | **FPIS** | **Distance from FPIS** | | | | | | | | | |
| --- | --- | --- | --- | --- | --- | --- | --- | --- | --- | --- | --- |
|  |  | **C1** | **C2** | **C3** | **C4** | **C5** | **C6** | **C7** | **C8** | **C9** | **C10** |
| **D1** | 0.2823 | 0.082 | 0.041 | 0.222 | 0.041 | 0.131 | 0.082 | 0.082 | 0 | 0.041 | 0.131 |
| **D2** | 0.4024 | 0.059 | 0.059 | 0.059 | 0.059 | 0.119 | 0.119 | 0.119 | 0 | 0 | 0.119 |
| **D3** | 0.3635 | 0.053 | 0 | 0.053 | 0.053 | 0.224 | 0.107 | 0.107 | 0.053 | 0.107 | 0.107 |
| **D4** | 0.3487 | 0 | 0.051 | 0.051 | 0 | 0.102 | 0.051 | 0.215 | 0.102 | 0.162 | 0.215 |
| **D5** | 0.3256 | 0.18 | 0 | 0 | 0.121 | 0.059 | 0.059 | 0.18 | 0.059 | 0.121 | 0.059 |
| **D6** | 0.4141 | 0 | 0.06 | 0.06 | 0.192 | 0.06 | 0.123 | 0.123 | 0 | 0 | 0.123 |
| **D7** | 0.3729 | 0.11 | 0.055 | 0.055 | 0.11 | 0.174 | 0.11 | 0.11 | 0 | 0.174 | 0.11 |
| **D8** | 0.3958 | 0.245 | 0.058 | 0 | 0.184 | 0.058 | 0.184 | 0.245 | 0 | 0.058 | 0.312 |
| **D9** | 0.4185 | 0 | 0.061 | 0.061 | 0.195 | 0 | 0.124 | 0.061 | 0 | 0.124 | 0.195 |
| **D10** | 0.3767 | 0.055 | 0 | 0.111 | 0.055 | 0.055 | 0.175 | 0.233 | 0.055 | 0.175 | 0.175 |
| **D11** | 0.3834 | 0 | 0.113 | 0.113 | 0.178 | 0.113 | 0.113 | 0.237 | 0 | 0.113 | 0.178 |
| **D12** | 0.3077 | 0.243 | 0.143 | 0.045 | 0.189 | 0.09 | 0.143 | 0.189 | 0 | 0.09 | 0.09 |
| **D13** | 0.3564 | 0.281 | 0.105 | 0.052 | 0.052 | 0.052 | 0.165 | 0.165 | 0 | 0.052 | 0.052 |
| **D14** | 0.3859 | 0.114 | 0.056 | 0 | 0.114 | 0.056 | 0.114 | 0.238 | 0.114 | 0.114 | 0.179 |
| **D15** | 0.3464 | 0.192 | 0.064 | 0 | 0.129 | 0.064 | 0.129 | 0.129 | 0 | 0.064 | 0.192 |
| **D16** | 0.0417 | 0.021 | 0.021 | 0.007 | 0.007 | 0.213 | 0.007 | 0.037 | 0 | 0.021 | 0.094 |
| **D17** | 0.0885 | 0.015 | 0 | 0.015 | 0.046 | 0.206 | 0.082 | 0.046 | 0 | 0.015 | 0.206 |
| **D18** | 0.2514 | 0.073 | 0.154 | 0.073 | 0.154 | 0.117 | 0.073 | 0.117 | 0 | 0.073 | 0.073 |
| **D19** | 0.3702 | 0.172 | 0.054 | 0.054 | 0.172 | 0.172 | 0.109 | 0.229 | 0 | 0.109 | 0.341 |
| **D20** | 0.317 | 0.046 | 0.147 | 0 | 0.046 | 0.25 | 0.046 | 0.25 | 0 | 0 | 0.196 |
| **D21** | 0.3821 | 0 | 0 | 0 | 0.056 | 0.302 | 0.113 | 0.056 | 0.056 | 0 | 0.056 |
| **D22** | 0.3866 | 0.056 | 0 | 0 | 0.056 | 0.056 | 0.18 | 0.114 | 0.056 | 0 | 0.056 |
| **D23** | 0.0755 | 0.039 | 0.013 | 0 | 0.07 | 0.013 | 0.039 | 0.013 | 0.013 | 0.039 | 0.178 |
| **D24** | 0.2762 | 0.218 | 0.17 | 0.218 | 0.128 | 0 | 0.081 | 0.218 | 0.128 | 0.128 | 0.17 |
| **D25** | 0.3349 | 0 | 0.049 | 0 | 0 | 0.156 | 0.099 | 0.156 | 0.049 | 0.099 | 0.207 |

**Table M. FNIS and distance of each alternative from FNIS.**

| **Code** | **FNIS** | **Distance from FNIS** | | | | | | | | | |
| --- | --- | --- | --- | --- | --- | --- | --- | --- | --- | --- | --- |
|  |  | **C1** | **C2** | **C3** | **C4** | **C5** | **C6** | **C7** | **C8** | **C9** | **C10** |
| **D1** | 0.0643 | 0.142 | 0.183 | 0 | 0.183 | 0.092 | 0.142 | 0.142 | 0.222 | 0.183 | 0.092 |
| **D2** | 0.2862 | 0.066 | 0.066 | 0.066 | 0.066 | 0 | 0 | 0 | 0.119 | 0.119 | 0 |
| **D3** | 0.142 | 0.174 | 0.224 | 0.174 | 0.174 | 0 | 0.118 | 0.118 | 0.174 | 0.118 | 0.118 |
| **D4** | 0.1366 | 0.215 | 0.167 | 0.167 | 0.215 | 0.113 | 0.167 | 0 | 0.113 | 0.056 | 0 |
| **D5** | 0.1468 | 0 | 0.18 | 0.18 | 0.061 | 0.123 | 0.123 | 0 | 0.123 | 0.061 | 0.123 |
| **D6** | 0.2257 | 0.192 | 0.134 | 0.134 | 0 | 0.134 | 0.074 | 0.074 | 0.192 | 0.192 | 0.074 |
| **D7** | 0.2034 | 0.067 | 0.121 | 0.121 | 0.067 | 0 | 0.067 | 0.067 | 0.174 | 0 | 0.067 |
| **D8** | 0.0884 | 0.071 | 0.257 | 0.312 | 0.129 | 0.257 | 0.129 | 0.071 | 0.312 | 0.257 | 0 |
| **D9** | 0.2277 | 0.195 | 0.136 | 0.136 | 0 | 0.195 | 0.075 | 0.136 | 0.195 | 0.075 | 0 |
| **D10** | 0.1469 | 0.181 | 0.233 | 0.122 | 0.181 | 0.181 | 0.061 | 0 | 0.181 | 0.061 | 0.061 |
| **D11** | 0.1493 | 0.237 | 0.125 | 0.125 | 0.063 | 0.125 | 0.125 | 0 | 0.237 | 0.125 | 0.063 |
| **D12** | 0.07 | 0 | 0.1 | 0.2 | 0.056 | 0.155 | 0.1 | 0.056 | 0.243 | 0.155 | 0.155 |
| **D13** | 0.0803 | 0 | 0.178 | 0.231 | 0.231 | 0.231 | 0.116 | 0.116 | 0.281 | 0.231 | 0.231 |
| **D14** | 0.1503 | 0.125 | 0.186 | 0.238 | 0.125 | 0.186 | 0.125 | 0 | 0.125 | 0.125 | 0.063 |
| **D15** | 0.1555 | 0 | 0.131 | 0.192 | 0.066 | 0.131 | 0.066 | 0.066 | 0.192 | 0.131 | 0 |
| **D16** | 0.2362 | 0.193 | 0.193 | 0.208 | 0.208 | 0 | 0.208 | 0.178 | 0.213 | 0.193 | 0.12 |
| **D17** | 0.2721 | 0.193 | 0.206 | 0.193 | 0.161 | 0 | 0.129 | 0.161 | 0.206 | 0.193 | 0 |
| **D18** | 0.0999 | 0.082 | 0 | 0.082 | 0 | 0.039 | 0.082 | 0.039 | 0.154 | 0.082 | 0.082 |
| **D19** | 0.0344 | 0.171 | 0.29 | 0.29 | 0.171 | 0.171 | 0.234 | 0.114 | 0.341 | 0.234 | 0 |
| **D20** | 0.0713 | 0.206 | 0.103 | 0.25 | 0.206 | 0 | 0.206 | 0 | 0.25 | 0.25 | 0.057 |
| **D21** | 0.0852 | 0.302 | 0.302 | 0.302 | 0.248 | 0 | 0.191 | 0.248 | 0.248 | 0.302 | 0.248 |
| **D22** | 0.2108 | 0.126 | 0.18 | 0.18 | 0.126 | 0.126 | 0 | 0.069 | 0.126 | 0.18 | 0.126 |
| **D23** | 0.2334 | 0.139 | 0.166 | 0.178 | 0.112 | 0.166 | 0.139 | 0.166 | 0.166 | 0.139 | 0 |
| **D24** | 0.0628 | 0 | 0.05 | 0 | 0.09 | 0.218 | 0.139 | 0 | 0.09 | 0.09 | 0.05 |
| **D25** | 0.131 | 0.207 | 0.161 | 0.207 | 0.207 | 0.054 | 0.109 | 0.054 | 0.161 | 0.109 | 0 |

**Table N. Relative closeness of each alternative/company and ranking.**

| **Company** | $\boldsymbol{o}_{\boldsymbol{i}}^{\boldsymbol{+}}$ | $\boldsymbol{o}_{\boldsymbol{i}}^{\boldsymbol{-}}$ | $\boldsymbol{C}_{\boldsymbol{i}}$ | **Rank** |
| --- | --- | --- | --- | --- |
| **C1** | 2.2535 | 3.2829 | 0.5929 | 5 |
| **C2** | 1.4732 | 4.0723 | 0.7343 | 3 |
| **C3** | 1.2494 | 4.2883 | 0.7744 | 2 |
| **C4** | 2.4072 | 3.1451 | 0.5664 | 6 |
| **C5** | 2.8426 | 2.6961 | 0.4868 | 8 |
| **C6** | 2.628 | 2.9219 | 0.5265 | 7 |
| **C7** | 3.6689 | 1.8752 | 0.3382 | 9 |
| **C8** | 0.6857 | 4.8402 | 0.8759 | 1 |
| **C9** | 1.8776 | 3.6609 | 0.6609 | 4 |
| **C10** | 3.8144 | 1.7293 | 0.3119 | 10 |
